# *Anaplasma phagocytophilum* subversion of host hepcidin-ferroportin iron nutritional immunity

**DOI:** 10.1128/mbio.01134-26

**Published:** 2026-06-15

**Authors:** Stephen L. Denton, Mingqun Lin, Elizabeta Nemeth, Yasuko Rikihisa

**Affiliations:** 1 Department of Veterinary Biosciences, College of Veterinary Medicine, Infectious Diseases Institute, The Ohio State University 198563, Columbus, Ohio, USA; 2 David Geffen School of Medicine, University of California at Los Angeles 12222, Los Angeles, California, USA; Massachusetts Institute of Technology, Cambridge, Massachusetts, USA

**Keywords:** *Anaplasma phagocytophilum*, ferroportin, iron transport, hepcidin

## Abstract

Obligatory intracellular bacterium *Anaplasma phagocytophilum* (*Aph*) causes an emerging infectious disease called human granulocytic anaplasmosis. Within host cells, *Aph* proliferates in a membrane-bound vacuole (*Aph*-vacuole) to which all required nutrients must be routed, including essential iron. Here, using a fluorescent labile iron-binding dye in live image analysis, we found that *Aph*-vacuoles are enriched with labile iron. Furthermore, ferroportin (Fpn), the only known iron exporter on the plasma membrane of host cells, increasingly encircled the *Aph*-vacuole post-invasion in a ring-like pattern. Fpn is regulated by the hormone hepcidin, which binds to Fpn and triggers its ubiquitination and internalization, thereby, inhibits iron availability to blood-borne pathogens. Fpn-GFP mutants deficient in ubiquitination (domain deletion or Y64H) did not encircle *Aph-*vacuoles. Fpn-GFP (D181V) and Fpn-GFP (D39A) mutants that are deficient in iron binding and transport could encircle *Aph-*vacuoles, but unlike Fpn-GFP (wild-type) did not increase labile iron in *Aph*-vacuoles or *Aph* growth. The gene encoding hepcidin is upregulated by inflammation. We found *Aph* infection induced the expression of the hepcidin mRNA and protein, and strong IL-6, IL-1β, and TNF-α mRNA expression by host cells. Altogether, these results suggest that *Aph* induces the production of proinflammatory cytokines for the upregulation of hepcidin which triggers the Fpn endocytosis and encasement of *Aph-*­vacuoles in a ubiquitination-dependent manner. Fpn internalization raises host cell cytoplasmic iron and may export iron into the vacuoles to enrich intravacuolar iron for the benefit of bacteria. This finding demonstrates pathogen manipulation of cellular iron export mechanisms and subversion of host hepcidin-Fpn iron nutritional immunity against pathogens.

**IMPORTANCE:**

Iron is an essential element for both humans and microorganisms, serving as a cofactor in key metabolic processes. Obligatory intracellular bacterium *Anaplasma phagocytophilum* infects and proliferates within a membrane-bound vacuole (*Aph-*vacuoles) of neutrophils and endothelial cells, competitively acquiring intracellular iron from the host. Upon exploration of cytoplasmic labile free iron levels and distribution in the *A. phagocytophilum*-infected and uninfected host cells, we uncovered the unique ability of this bacterium to enrich intravacuolar iron. Ferroportin is the only cellular iron exporter of host cells, which is regulated by the hepatic hormone hepcidin. Herein, we investigate the role of ferroportin and endogenous hepcidin locally produced by infected host cells for iron enrichment in *Aph-*vacuoles. Ultimately, this study provides new insights into the novel mechanisms of microbial manipulation of host cells to acquire the essential micronutrient iron and overcoming host nutritional immunity, which may facilitate more effective treatment and prevention.

## INTRODUCTION

Rickettsioses including Anaplasmosis and Ehrlichiosis are among the most deadly vector-borne infectious diseases and greatly increasing in worldwide prevalence (1). Rickettsiae are obligatory intracellular bacteria that infect blood and endothelial cells. Human granulocytic anaplasmosis (HGA), caused by infection with *Anaplasma phagocytophilum* (*Aph*), is second in prevalence only to Lyme disease (2, 3), and human monocytic ehrlichiosis (HME), caused by *Ehrlichia chaffeensis* (*Ech*), is second in fatality only to Rocky Mountain spotted fever among tick-borne diseases in the US (4). *Aph* infects and replicates inside granulocytes (2, 5), and *Ech* infects and replicates inside monocytes-macrophages (6, 7). HGA and HME are severe flu-like febrile diseases accompanied by hematologic abnormalities and signs of hepatitis. There are no FDA-approved vaccines to protect against rickettsial diseases including HGA and HME. The only current therapy for HGA and HME is the broad-spectrum antibiotic doxycycline, which is generally effective with sufficiently early treatment, but no alternatives exist if cases are not treated early, or if microbial resistance develops.

Iron is an essential element for most living organisms, serving as a cofactor in key metabolic processes. As excess iron is toxic, however, systemic and intracellular iron levels are tightly regulated in animals by cellular iron import, storage, and export mechanisms (8, 9). Furthermore, sequestering iron from invading pathogens is an important arm of innate immunity, referred to as nutritional immunity (8, 10, 11). Cellular iron nutritional immunity is exemplified by the divalent cation transporter NRAMP1, that exports Fe^2+^ from late endosomes/lysosomes, driving resistance to facultative intracellular pathogens that occupy these niches (such as *Salmonella, Leishmania*, and *Mycobacterium*) by limiting pathogen growth (12–14). The known systemic iron nutritional immunity is hypoferremia mediated by peptide hormone hepcidin (Hepc, secreted mainly by liver), which not only deprives access to iron to blood-borne pathogens directly but also shapes iron-dependent host cellular pathways that pathogens rely upon or defend against (such as glucose metabolism, oxidative stress, and immune regulation) (15). Infectious stimuli with pathogens induce hepatocytes to produce and secrete Hepc into the blood (16). When systemic Hepc reaches cells, it occludes ferroportin (Fpn), preventing iron export, but also inducing ubiquitination of the intracellular domain, which triggers a Fpn internalization and degradation cascade (17–20). As such, the net iron export is reduced, preventing outgrowth of many invading extracellular pathogens (16, 21, 22). Even though Fpn remains the only known cellular iron exporter of the human host for *Aph* (23, 24), it’s impact on nutritional immunity to obligate intracellular pathogens like *Aph* is unknown.

For rickettsiae, iron acquisition is essential, but it is mostly unknown how these bacteria acquire iron from the host intracellular environment, and how they overcome host iron-sequestering nutritional immunity. The labile iron pool (LIP) is the pool of Fe^2+^ iron in a cell that is not bound to storage or carrier molecules. It is a crucial but transient intermediate in cellular iron metabolism, serving as a source of iron for metabolic processes. While many other bacteria use siderophores, high-affinity iron-chelating compounds that competitively capture iron from the LIP or protein-bound sources (9), all rickettsiae members lack siderophores. For intracellular pathogens *Salmonella, Mycobacterium,* and *Leishmania donovanii,* a strategy is to alter iron flow *via* influencing expression levels and compartmental distribution of iron transporters like NRAMP1, DMT1, and Fpn (25–29). *Ech* replicates in an early endosome-like compartment (vacuole) that does not fuse with lysosomes (30, 31). We previously discovered *Ech* acquires iron from two distinct mammalian iron-binding proteins: transferrin (Tf) and ferritin. During initial *Ech* infection, Tf receptor (TfR) is upregulated and recruits endocytosed iron-laden Tf to the *Ech-*vacuole (32–34). Upon establishment of infection, secretion of *Ech* effector Etf-3 induces ferritinophagy, the autophagic liberation of iron from ferritin for bacterial uptake (35).

*Aph* is closely related to *Ech,* sharing 98% of protein coding-genes, and it requires iron (36). However, *Aph* replicates in the membrane-bound compartment, the *Aph*-vacuole, bearing markers of early autophagosomes and ER-Golgi exit sites and lacking endosome-lysosome markers (30, 31, 37, 38). *Aph* does not use *Ech’s* iron-acquisition mechanisms, as unlike *Ech*, (i) *Aph* infection does not upregulate TfR mRNA (32); (ii) *Aph-*containing vacuoles do not acquire Tf or TfR (30); and (iii) *Aph* lacks Etf-3 orthologs that cause ferritinophagy. We, therefore, investigated *Aph* iron uptake mechanisms by examining LIP distribution and levels, and host iron transport systems to create wider mechanistic understanding of iron-acquisition strategies used by rickettsial pathogens and enable developing new effective countermeasures.

## RESULTS

### Labile cellular iron is enriched in *Aph-*vacuoles, and ferritin is upregulated by *Aph* infection

**Fig 1:**
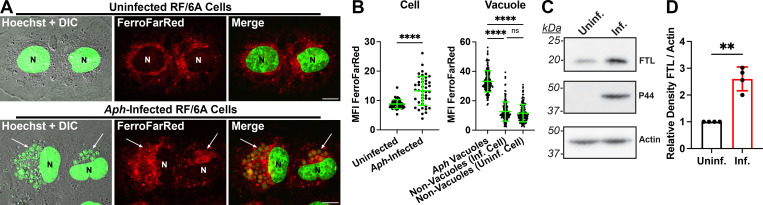
Labile iron pool is enriched in *Aph-*vacuoles, and cellular ferritin is upregulated in *Aph*-infected cells. (**A**) LIP (Labile iron pool, red) detected with FerroFarRed dye in uninfected and *Aph*-infected RF/6A cells 1 dpi. Live cell image under a Leica Thunder microscope. DNA of nucleus (N) and *Aph* was stained with Hoechst dye (pseudocolored green). Small green globules indicated by white arrows are *Aph*-vacuoles. Merge, superimposed fluorescence images. DIC, differential interference contrast. Bar, 10 µm. (**B**) Scatter plots showing MFI (Mean Fluorescence Intensity) in arbitrary units in individual uninfected and *Aph*-infected cells, vacuoles, and randomly paired non-vacuole areas (*n* = 43 cells/group, from which 153 vacuoles were assessed). Visualization of analysis method depicted in Fig. S1. Mean ± SD assessed by Student’s *t*-test (left graph) or One-Way ANOVA (right graph) where **** indicates *P* < 0.0001, ns, not significant. (**C**) Western blot analysis of uninfected (Uninf.) and *Aph*-infected (Inf. 2 dpi) HL-60 cells with anti-ferritin light chain (FTL), *Aph* P44, and actin antibodies. (**D**) Band density ratios of FTL relative to actin. The ratio of uninfected HL-60 cells was arbitrarily set to 1. Mean ± SD where ** indicates significantly different by student’s *t*-test (*P* < 0.01). Images and data are representative of three independent experiments.

*Aph* infects neutrophils, other granulocytes, and endothelial cells *in vivo* (39). Monkey endothelial cell line RF/6A is permissive to *Aph* infection (40) and used for unambiguous cellular localization studies, as they are flat and thinly spread adherent cells (41–43). As *Aph* replicates in membrane-bound vacuoles, we examined LIP distribution and relative amounts in RF/6A cells by using FerroFarRed probe (35, 44, 45), which is membrane-permeable and fluoresces when N-oxide is selectively deoxygenized by Fe^2+^ in live cell fluorescence microscopy. *Aph* vacuoles were defined by the presence of *Aph* by Hoechst fluorescence which binds bacterial DNA, as membrane permeabilization for immunolabeling of intracellular *Aph-*vacuoles hinders live image analysis. ImageJ was used to quantitate fluorescence intensity in the area of a defined Region of Interest (ROI) (Fig. S1). The *Aph*-vacuoles were enriched for LIP (Fig. 1A). This was indicated by the significant enrichment of FerroFarRed fluorescence of the *Aph*-vacuolar area compared to non-*Aph* sites in uninfected or infected cells (Fig. 1B). Consequently, relative LIP fluorescence was significantly increased in *Aph*-infected cells than in uninfected cells (Fig. 1B). The increased LIP may increase intracellular ferritin levels because ferritin stores excess of iron within a cell (46). Ferritin levels were determined by western blot analysis of ferritin light chain (FTL) normalized by human actin, with *Aph* outer membrane protein P44 (47) as an infected lane control. In *Aph*-infected HL-60 cells, ferritin was significantly increased compared to uninfected cells (Fig. 1C and D). As described in Introduction, these findings further confirm the distinct nature of *Aph*-infected cells vs *Ech*-infected cells, wherein the latter ferritin is diminished due to ferritinophagy (35). Accordingly, enrichment of iron within *Aph-*vacuoles points to a unique mechanism of *Aph* infection on iron redistribution and homeostasis within host cells.

### Fpn-GFP and endogenous Fpn localize on *Aph*-containing vacuoles

**Fig 2:**
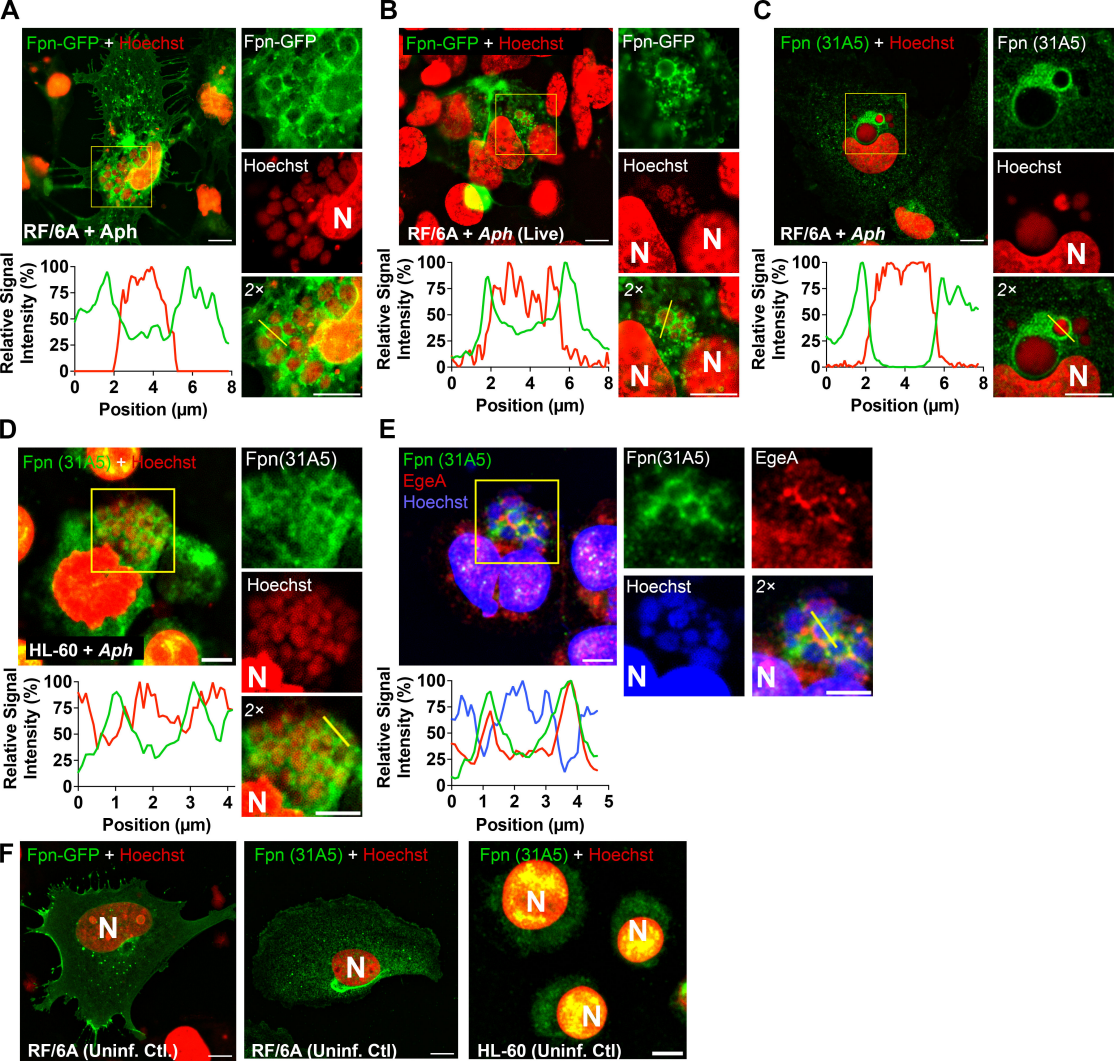
Fpn-GFP and endogenous Fpn localize to *Aph*-containing vacuoles. (**A**) Fpn-GFP (green)-transfected RF/6A cells were infected with host cell-free *Aph* at 1 dpt. At 3 dpt/2 dpi, cells were fixed and host and bacterial DNA were stained with Hoechst (pseudocolored red). Merge, merge of two fluorescence images. N, nucleus. (**B**) RF/6A cells were transfected with Fpn-GFP (green) and infected with *Aph* at 2 dpt. Live cell DNA staining was performed with Hoechst (pseudocolored red), and cells were imaged at 1.5 dpi/3.5 dpt under 37°C and 5% CO_2_ conditions. (**C**) Untransfected, *Aph-*infected RF/6A cells were fixed at 2 dpi, permeabilized, and immunostained with mouse monoclonal anti-human Fpn (31A5, green) and Hoechst. (**D**) *Aph*-infected HL-60 cells were fixed at 2 dpi, permeabilized, and stained with anti-Fpn (31A5, green) and Hoechst. (**E**) *Aph-*vacuoles of HL-60 cells at 2 dpi, permeabilized, and stained with anti-Fpn (31A5, green), with anti-*Aph* EgeA (red), and Hoechst (blue). (**A–E**) boxed area is enlarged 2 × on the right of the respective panel with individual channels and merged image. Relative fluorescence signal intensity plots along the yellow lines in the enlarged merge images show DNA (red line) (**A–D**), and transfected Fpn-GFP (green line) (**A and B**) or immunofluorescence-labeled endogenous Fpn (green line) (**C–D**), showing Fpn peaks encasing bacteria DNA peak in the vacuoles but not overlapping, and (**E**) EgeA (red line) peaks overlapping with Fpn (green line) on the *Aph-*vacuole encasing bacterial DNA (blue line) peak. (**F**) Localization of Fpn-GFP at 2 dpt in uninfected RF/6A cells (Uninf. Ctl), or Fpn (31A5) immunofluorescence in uninfected RF/6A and HL-60 cells (Uninf. Ctl). Scale bars: 10 µm in RF/6A cells; 5 µm in HL-60 cells. Images are representative of three independent experiments.

Because *Aph-*vacuoles were relatively iron dense and elevated LIP levels in infected cells, we hypothesized that infection may change distribution of host iron transport machinery. TfR, a primary mediator of iron import, localizes to *Ech*-vacuoles, but not to *Aph-*vacuoles (32), whereas Fpn in the plasma membrane (PM) is the sole cellular Fe^2+^ exporter for the host, and reduced PM localization of Fpn leads to an increase in LIP in uninfected cells (48). Human promyelocytic leukemia cell line HL-60 is widely used for *Aph* propagation, and infection studies, but difficult to transfect (2, 49), whereas RF/6A cells have higher transfection efficincy. Therefore, we analyzed intracellular distribution of endogenous Fpn in HL-60 cells and RF/6A cells and Fpn-GFP in transfected RF/6A cells, with and without *Aph* infection. We observed distinct localization of Fpn-GFP encircling individual *Aph-*vacuoles in RF/6A cells after chemical fixation or by live-cell image analysis (Fig. 2A and B). Endogenous Fpn spatial distribution was examined by immunofluorescence (IF) labeling (after chemical fixation and membrane permeabilization) with the 31A5 antibody that can recognize native human Fpn (50). In *Aph-*infected RF/6A cells (Fig. 2C) and HL-60 cells (Fig. 2D), endogenous Fpn was also found surrounding individual *Aph* vacuoles. In each case, line scan results spanning *Aph-*vacuoles showed that Fpn fluorescence (either GFP-tagged or IF-labeled) peaked on both sides of the Hoechst fluorescence (bacterial DNA) peak (Fig. 2A through D). To demonstrate Fpn localization at the *Aph-*vacuole surface, we examined endogenous Fpn localization with *Aph* type IV secretion system effector ER-Golgi exit protein of *Anaplasma* (EgeA), which localizes to the *Aph-*vacuole surface (38). In *Aph-*infected HL-60 cells, endogenous Fpn, indeed, colocalized with native EgeA (Fig. 2E; Fig. S2 ). Fpn-GFP was primarily localized on PM in uninfected RF/6A cells, whereas IF labeling of endogenous Fpn in uninfected RF/6A and HL-60 cells showed more homogeneous distribution in the cytoplasm rather than on PM (Fig. 2F). Normal mouse serum control of infected and uninfected cells under the same staining condition did not show non-specific labeling as shown previously (38).

### Fpn-GFP encircling *Aph-*vacuoles occurs subsequent to bacterial invasion and requires bacterial protein synthesis

**Fig 3:**
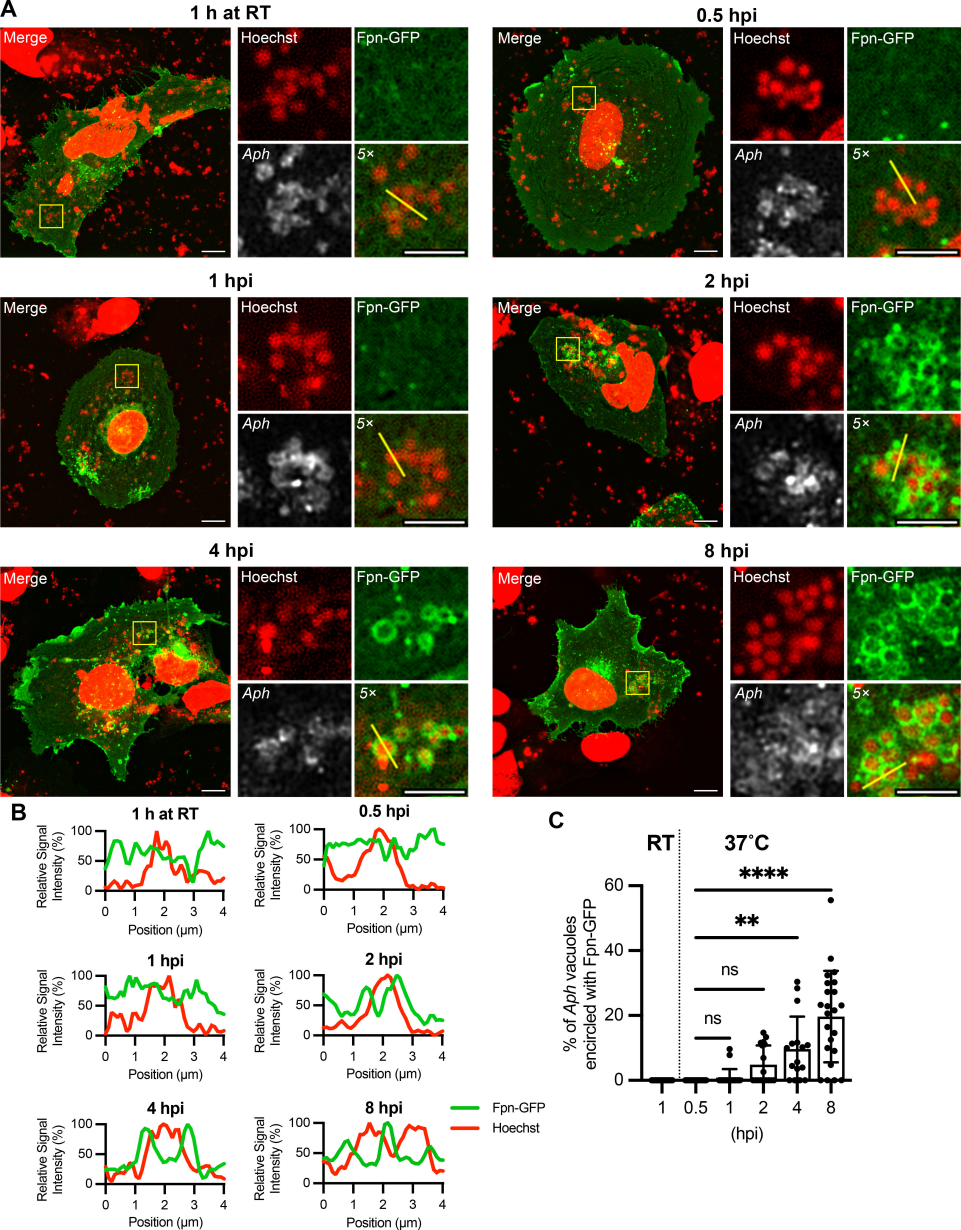
Fpn-GFP localization to *Aph* vacuoles occurs subsequent to bacterial entry. (**A**) Host cell-free *Aph* was added to Fpn-GFP-transfected RF/6A cells at 2 dpt at room temperature (RT) to allow only binding, or at 37°C to allow binding and internalization, and harvested at indicated hpi. *Aph* DNA was labeled with Hoechst (pseudocolored red) and *Aph* was labeled with anti-*Aph* (pseudocolored white). Merge, merge of Hoechst and Fpn-GFP images (bar, 10 µm). Each boxed area is enlarged 5 × on the right (bar, 5 µm). (**B**) Relative fluorescence signal intensity profiles between Fpn-GFP (green), and *Aph* DNA (red) along the yellow line in enlarged merged image in A, show the Fpn peaks are encasing bacteria DNA peak within the vacuoles starting at 2 hpi. (**C**) Percentage of vacuoles encased with Fpn-GFP per cell. >800 vacuoles or bacteria were counted across at least 20 cells at each time point. One-way ANOVA ****P < 0.0001, **P < 0.01, ns, not significant. Images are representative of three independent experiments.

**Fig 4:**
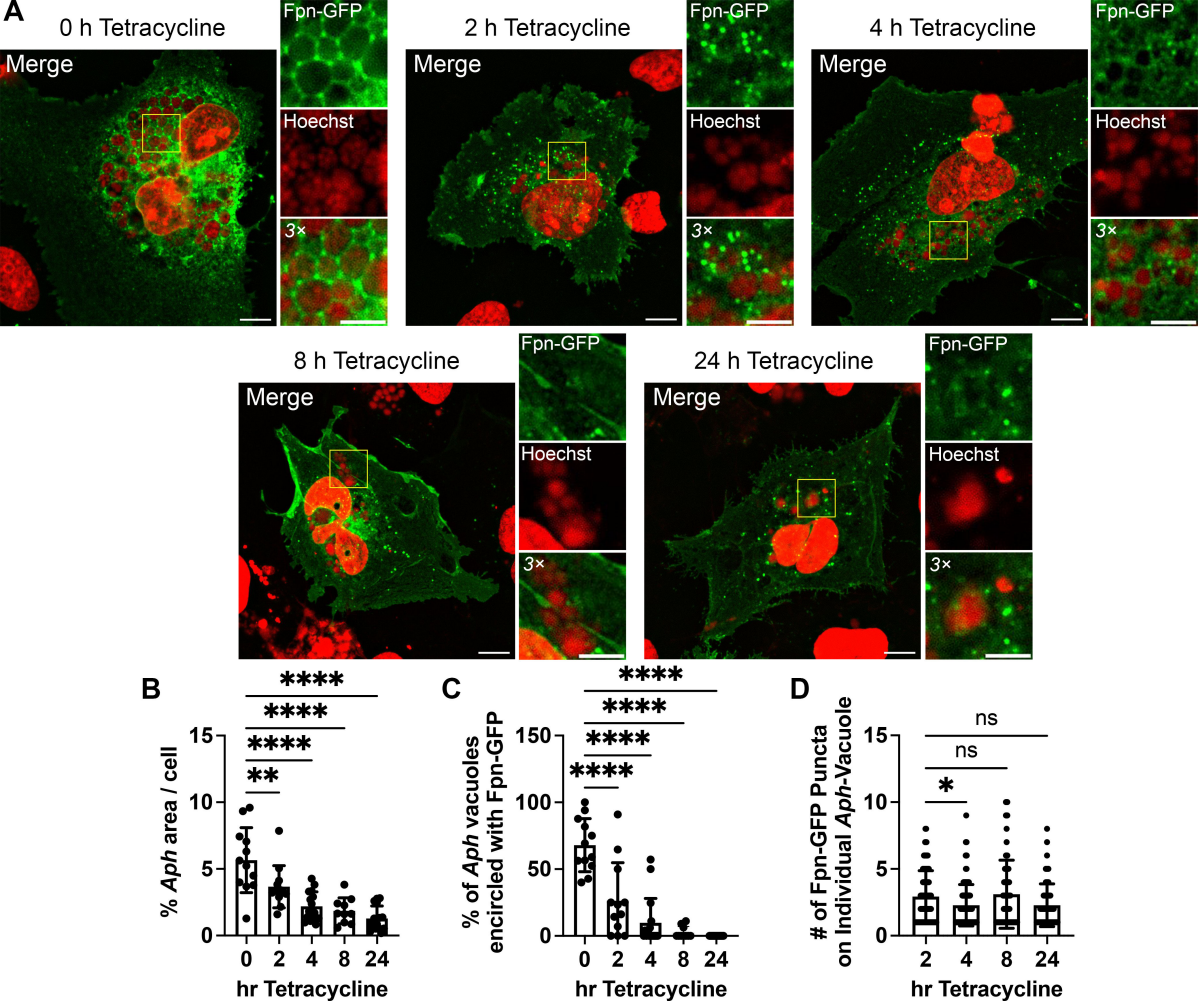
Fpn-GFP localization to *Aph-*vacuoles is dependent on *Aph* protein production. (**A**) RF6/A cells were transfected with Fpn-GFP, infected with host cell-free *Aph* (MOI: 250), and treated with 5 µg/mL tetracycline for the indicated periods immediately proceeding harvest at 3 dpt/2 dpi. Host and *Aph* DNA, labeled with Hoechst (pseudocolored red), and Fpn-GFP (green) fluorescence were imaged. Merge, merge of Hoechst and Fpn-GFP images (bar, 10 µm). Each boxed area is enlarged 3 × on the right (bar, 5 µm). (**B**) % of *Aph* infection determined by area of *Aph* (µm^2^)/area of Fpn-GFP expressing host cell (µm^2^) *100. *N* = 10–16 cells per treatment period. (**C**) % of *Aph* vacuoles with circling, non-fragmented Fpn-GFP rings scored in ImageJ. >150 *Aph-*vacuoles were counted from 10 cells per treatment period. (**D**) Number of Fpn-GFP puncta on individual *Aph*-vacuoles. ≥60 *Aph-*vacuoles were counted from 10 cells per treatment period. One-way ANOVA, ns, not significant; *P < 0.05, **P < 0.01, ****P < 0.0001. Data representative of two independent experiments.

Fpn-GFP and endogenous Fpn encircling *Aph-*vacuoles were evident at exponential to stationary stages of bacterial proliferation (Fig. 2). At this stage, Fpn may appear compressed between crowded *Aph-*vacuoles (Fig. 2). Next, we examined early time course of Fpn localization when much less *Aph* is present, and determined when the encircling occurs. First, to determine if Fpn is recruited to the site of bacterial binding to PM, we incubated *Aph* and Fpn-GFP-transfected RF/6A cells at room temperature. Under this condition, *Aph* bound to the host cells, but could not internalize, and there were no instances of PM Fpn-GFP colocalization with bound *Aph* (Fig. 3). At endocytosis-permissive 37°C, *Aph* began to be internalized (Fig. 3), but Fpn-GFP did not encircle a significant proportion of *Aph-*vacuoles until 2 h post infection (hpi). Thereafter, increasingly higher proportions of *Aph-*vacuoles were encircled by Fpn-GFP in a distinct ring-like pattern (Fig. 3). To analyze if encasement of *Aph*-vacuoles with Fpn-GFP is dependent on active protein synthesis by intracellular *Aph,* we next assessed effects of bacterial protein synthesis inhibitor tetracycline on Fpn-GFP localization starting at 2 days post infection (dpi). Starting at 2 h of treatment, significant reduction of *Aph* vacuoles occurred (Fig. 4A and B). Concurrently, Fpn-GFP encasement of the *Aph-*vacuoles was diminished or lost with tetracycline treatment (Fig. 4A) in a treatment time-dependent manner (Fig. 4C). Additionally, there were punctate patterns of Fpn-GFP observed near or on *Aph-*vacuoles, suggestive of endosomal/lysosomal Fpn vesicles or vestiges thereof (17, 51). The abundance of these puncta on individual *Aph-*vacuoles was not significantly different during 2–24 h tetracycline treatment (Fig. 4D). Taken together, these results indicate that both active intracellular bacteria and infection-induced host cell signaling must occur to induce Fpn encasement of *Aph* vacuoles.

### Fpn ubiquitination motifs are necessary for Fpn-GFP localization to *Aph-*vacuoles

**Fig 5:**
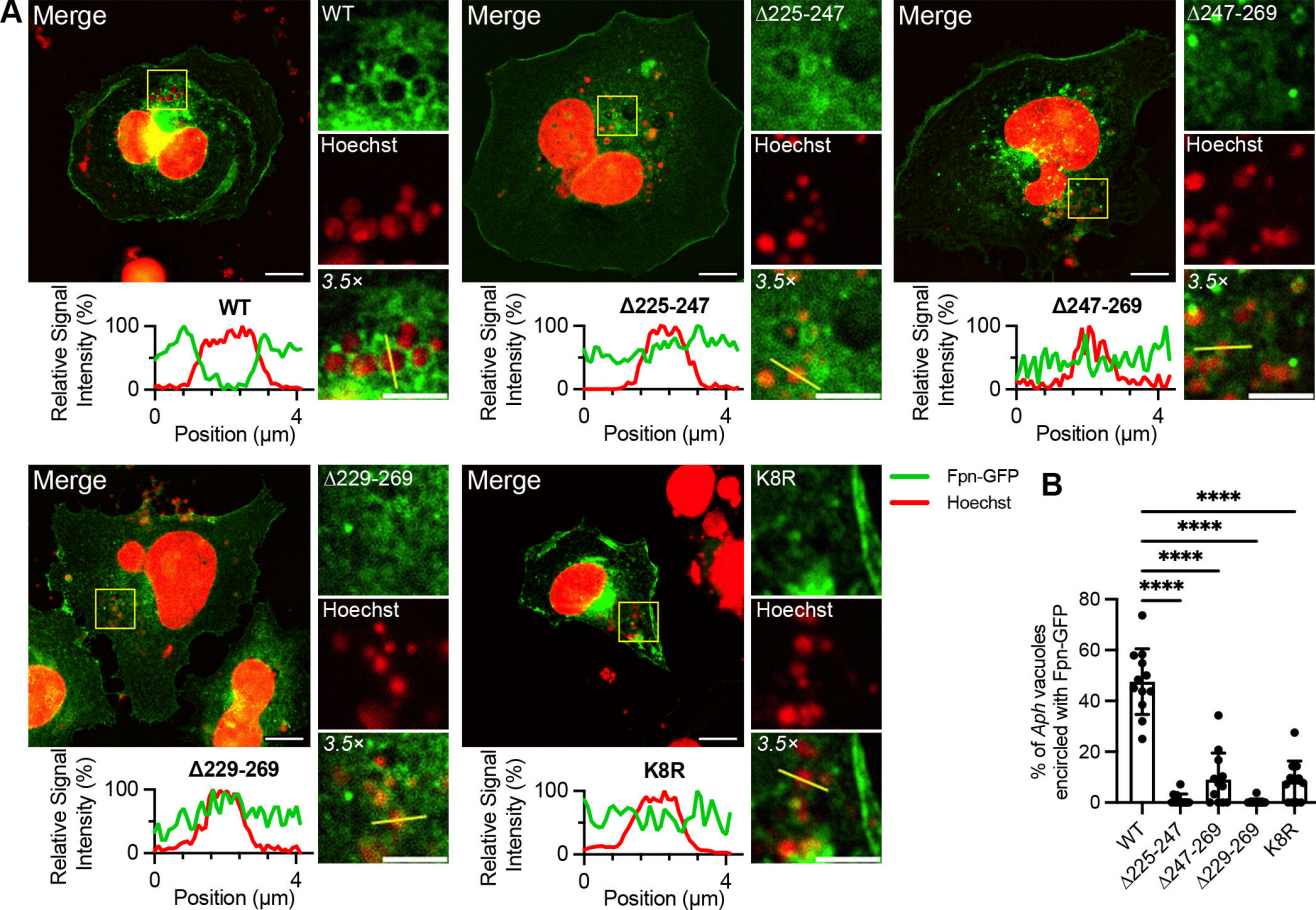
Fpn-GFP motifs for ubiquitination are required for Fpn-GFP localization to *Aph-*vacuoles. All lysine residues of Fpn-GFP (K8R) between positions 225–269 are substituted for arginine. This mutant along with ∆229–269, ∆225–247, and ∆247–269 are deficient in Hepc-induced ubiquitination. (**A**) *Aph* was added to Fpn (WT or indicated mutant)-GFP (green)-transfected RF/6A cells at 2 dpt, and cells were harvested at 8 hpi. *Aph* DNAs were labeled with Hoechst (pseudocolored red). Merge, merge of Hoechst and Fpn-GFP images (bar, 10 µm). Each boxed area is enlarged 3.5 × on the right (bar, 5 µm). Relative fluorescence signal intensity profiles between Fpn-GFP (green), and *Aph* DNA (red) along the yellow line in enlarged merged image, showing the Fpn peaks and bacteria DNA peak. (**B**) Percentage of *Aph*-vacuoles encased with Fpn-GFP. >200 vacuoles were scored in at least in 12 cells per condition. One-way ANOVA, ****P < 0.0001. Data are representative of three independent experiments.

**Fig 6:**
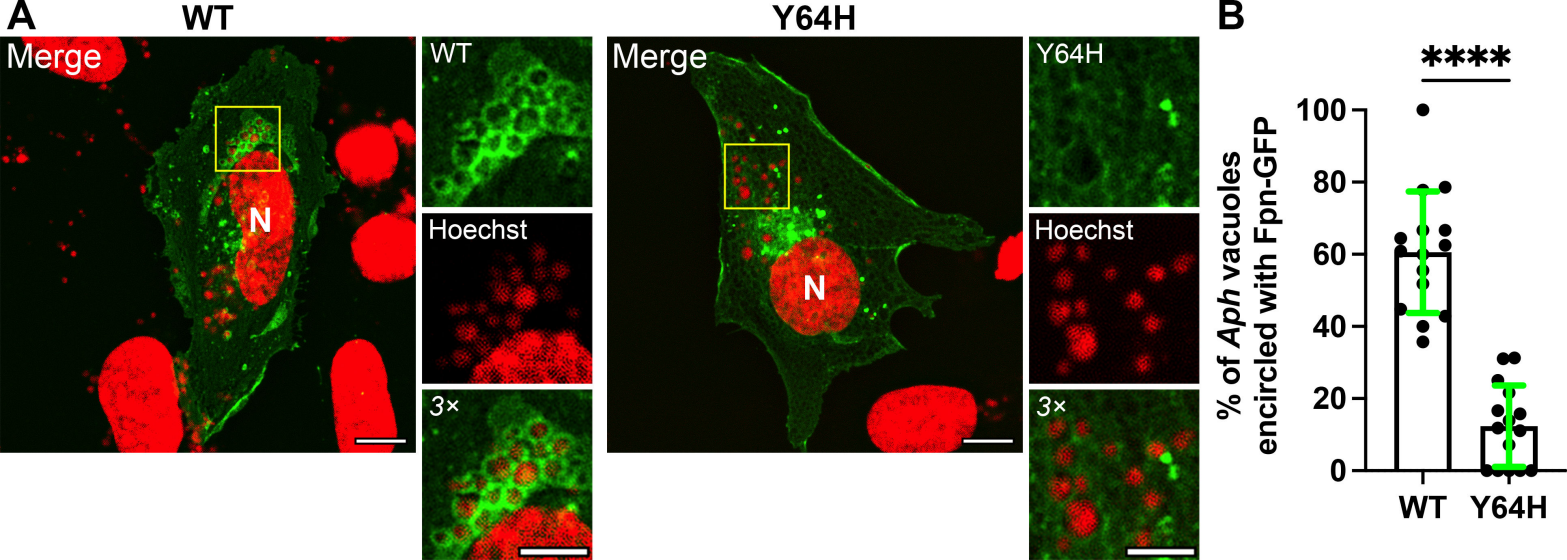
Fpn-GFP (Y64H), which binds Hepc but cannot internalize, does not localize to *Aph-*vacuoles. (**A**) *Aph* was added to Fpn (WT or Y64H)-GFP (green)-transfected RF/6A cells at 2 dpt, and cells were harvested at 12 hpi. *Aph* and host DNAs were labeled with Hoechst (pseudocolored red). N, Nucleus. Merge, merge of Hoechst and Fpn-GFP images (bar, 10 µm). Boxed areas are enlarged 3 × on the right (bar, 5 µm). (**B**) Percentage of *Aph*-containing vacuoles with encircled Fpn-GFP was scored by Image J. > 400 *Aph-*vacuoles were counted from 15 cells per condition. Student’s *t*-test, ****P < 0.0001. Data representative of two independent experiments.

To investigate if PM Fpn internalization is required for Fpn localization to *Aph*-vacuoles, we used Fpn mutants: Fpn (∆229–269)-GFP, Fpn (∆225–247)-GFP, and Fpn (∆247–269)-GFP, which lack the intracellular domain between positions 225–290 (Fig. S3) and are expressed on PM, but cannot internalize in response to Hepc (20). Each of these mutant plasmids had similar transfection efficiencies and expression levels (Fig. S4), yet, our results showed Fpn mutants significantly less frequently encircled *Aph-*vacuoles than WT (wild-type) Fpn (Fig. 5). The lysine residues in Fpn residue 225–290 (Fig. S3) are the sites for ubiquitination and are required for Hepc-induced internalization (18). Thus, we used Fpn(K8R)-GFP (18) to test if these lysine are required for Fpn localization to *Aph*-vacuoles, which showed significant less Fpn(K8R) mutant encircling of *Aph*-vacuoles (Fig. 5). Lastly, Fpn(Y64H) is a natural point mutation in humans that binds Hepc (18, 52). Fpn(Y64H)-GFP can export iron, but upon Hepc treatment, it is not ubiquitinated nor internalized (19, 53). Fpn (Y64H)-GFP encircled *Aph-*vacuoles significantly less than WT Fpn (Fig. 6). These results altogether suggest that Hepc-induced Fpn internalization pathway is required for Fpn localization to *Aph*-vacuoles.

### Iron binding/transport deficient mutants do not increase LIP in *Aph-*vacuoles or *Aph* replication

**Fig 7:**
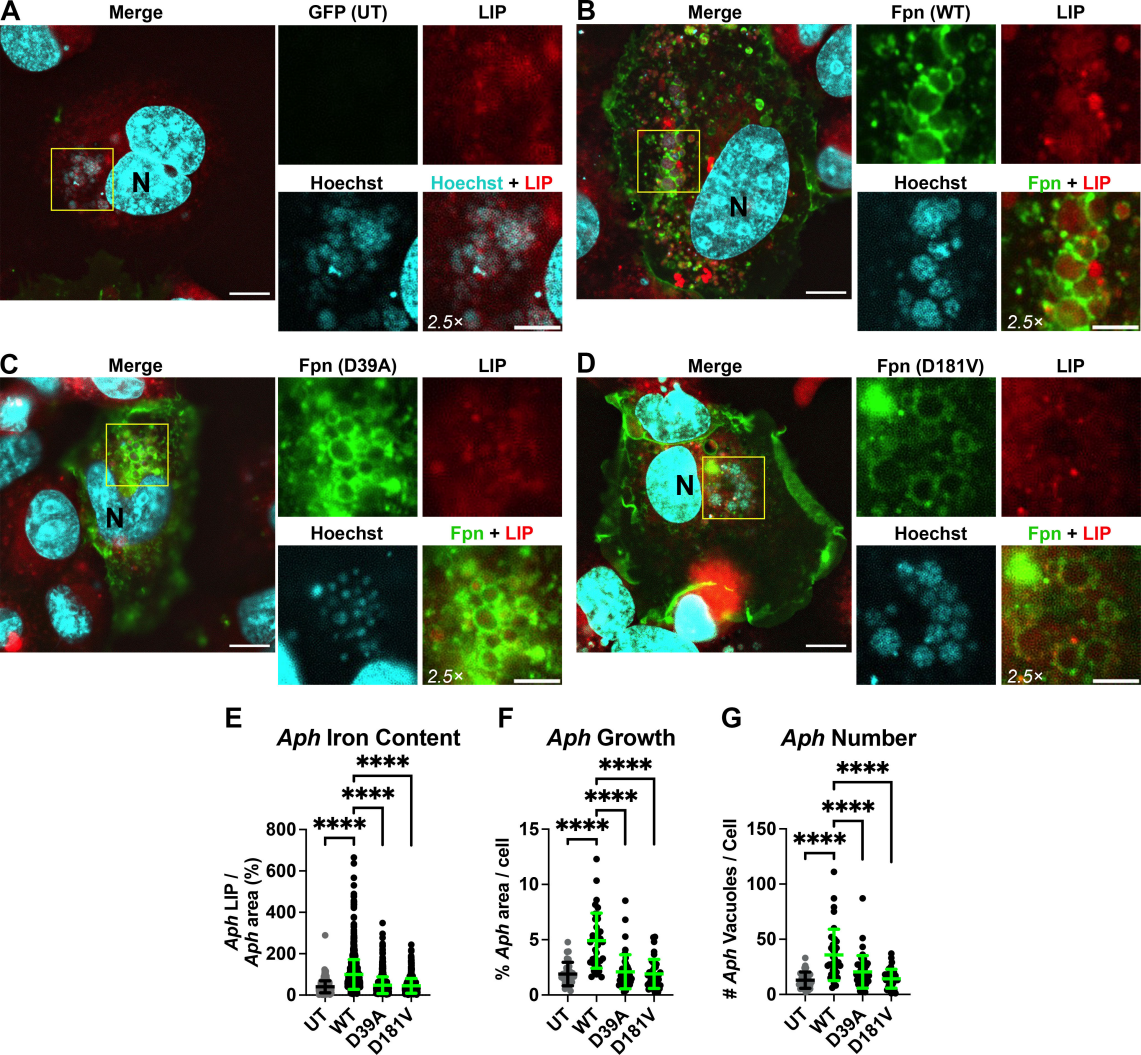
Iron binding/transport deficient mutants do not increase LIP in *Aph-*vacuoles or *Aph* replication RF/6A cells were transfected with plasmids encoding WT, D39A, or D181V Fpn-GFP (colored green) and infected with host cell-free *Aph.* At 1.5 dpi and 3.5 dpt, cells were dyed with FerroFarRed to detect LIP (colored red) and Hoechst 33342 dye (pseudo-colored cyan) for the detection of host and *Aph* DNAs. Live cells were imaged under a Leica DMi8 Thunder microscope at 37°C. (**A**) Un-transfected (UT), (**B**) Transfected with Fpn-GFP (WT), (**C**) Transfected with Fpn-GFP (D39A), or (**D**) Transfected with Fpn-GFP (D181V). Merge, merge of LIP, Hoechst, and indicated Fpn-GFP mutant (bar, 10 µm). Yellow-boxed areas are enlarged 2.5 × on the right (bar, 5 µm). *Aph-*vacuole boundaries determined in ImageJ by globular, non-nuclear Hoechst fluorescence. (**E**) Iron content measured by LIP MFI of vacuoles normalized by the *Aph* area % of the infected cell. *N* = 541–1,324 vacuoles. (**F**) % of *Aph* area/cell determined by area (µm^2^) of *Aph-*vacuoles/area (µm^2^) of GFP-expressing cell * 100. *N* = 30–45 cells. (**G**) Total *Aph*-vacuole numbers were enumerated in GFP-expressing cells for each plasmid type. *N* = 30–45 cells. Statistics: One-Way ANOVA to WT control, ***P < 0.001, ****P < 0.0001. Data representative of three independent experiments.

Fpn’s iron export and Hepc-binding abilities are closely linked, but residues that are most important for iron binding/export and independent from Hepc binding are D39 and D181 (Fig. S3 and S5) (54–56). We, thus, tested if Fpn (D39A)-GFP or Fpn (D181V)-GFP can encircle *Aph-*vacuoles, and increase vacuolar LIP, and/or promote *Aph* growth vs Fpn (WT)-GFP. The results showed both Fpn (D39A)-GFP and Fpn (D181V)-GFP can encircle *Aph-*vacuoles, like Fpn (WT)-GFP (Fig. 7B through D). Fpn (WT)-GFP enhanced relative LIP levels in *Aph-*vacuoles/host cell cytoplasm compared to *Aph-*vacuoles of un-transfected cells, but not in Fpn (D39A)- or Fpn (D181V)-transfected cells (Fig. 7B through E). In turn, *Aph* growth within WT-expressing cells was greater than in un-transfected cells, or Fpn (D39A)- or Fpn (D181V)-transfected cells (Fig. 7F and G), suggesting that not only encircling *Aph*-vacuoles, but also the ability of Fpn to transport iron is required to enrich intravacuolar LIP and enhance *Aph* growth.

### *Aph* infection induces production of autologous *HAMP* mRNA and elevates Hepc protein levels

**Fig 8:**
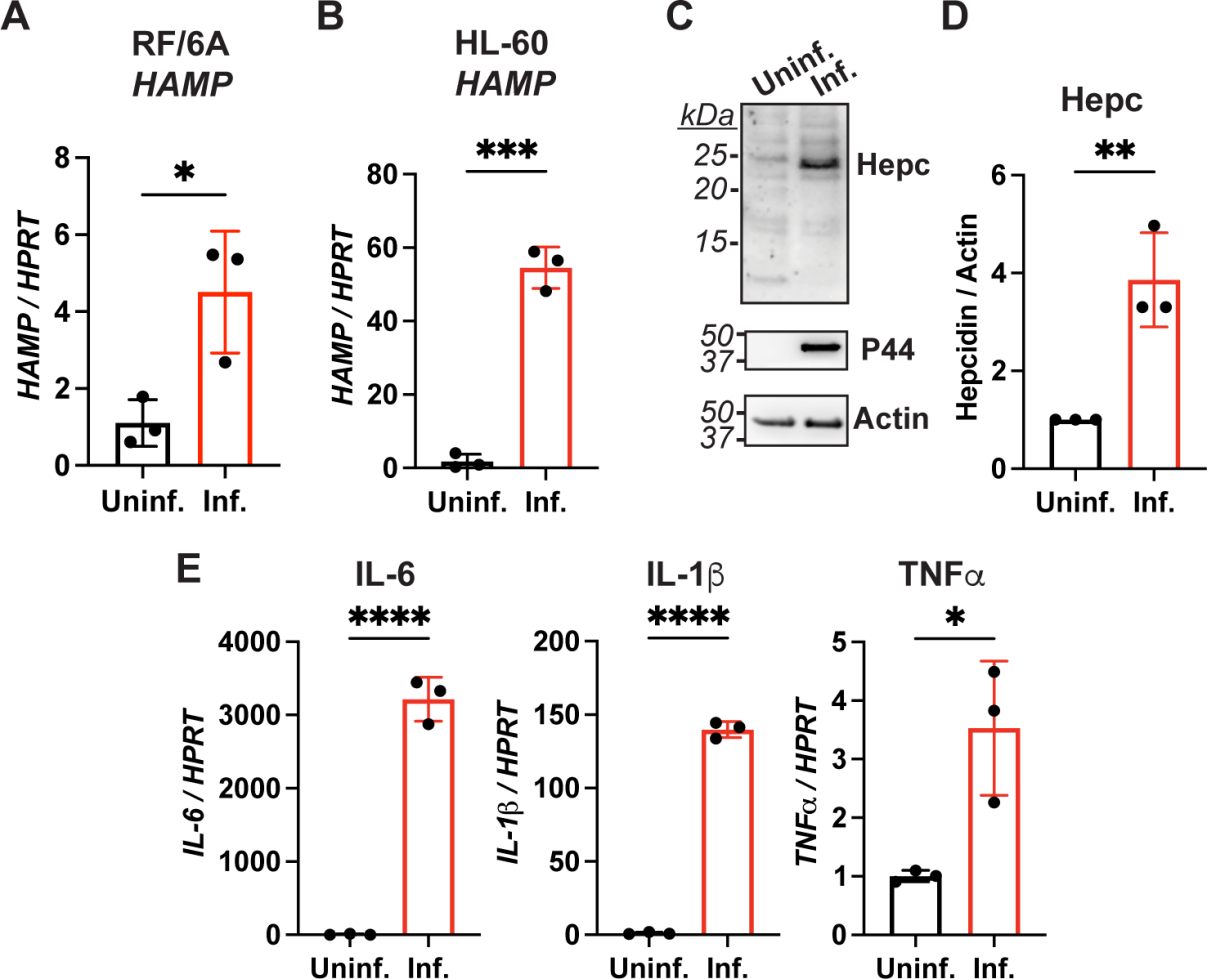
*Aph* infection induces production of Hepc by the host cells. (**A and B**) Relative *HAMP* mRNA levels normalized to human *HPRT* mRNA (∆∆Ct) in uninfected and *Aph*-infected (A) RF/6A cells at 48 hpi, or (B) HL-60 cells at 12 hpi. (**C**) Lysates from uninfected and *Aph*-infected HL-60 cells at 2 dpi were analyzed by western blotting using antibodies against human Hepc, human actin, and *Aph* P44. (**D**) The relative band density of Hepc was normalized against actin with the level of the uninfected group set as 1. (**E**) Relative mRNA abundance of human TNF⍺, IL-6, and IL-1β from samples normalized to human HPRT in HL-60 cells as in (**B**) by the ∆∆Ct method. Data represent the mean ± SD from three independent experiments. Student’s *t*-test, *P < 0.05; **P < 0.01; ***P < 0.001; ****P < 0.0001.

**Fig 9:**
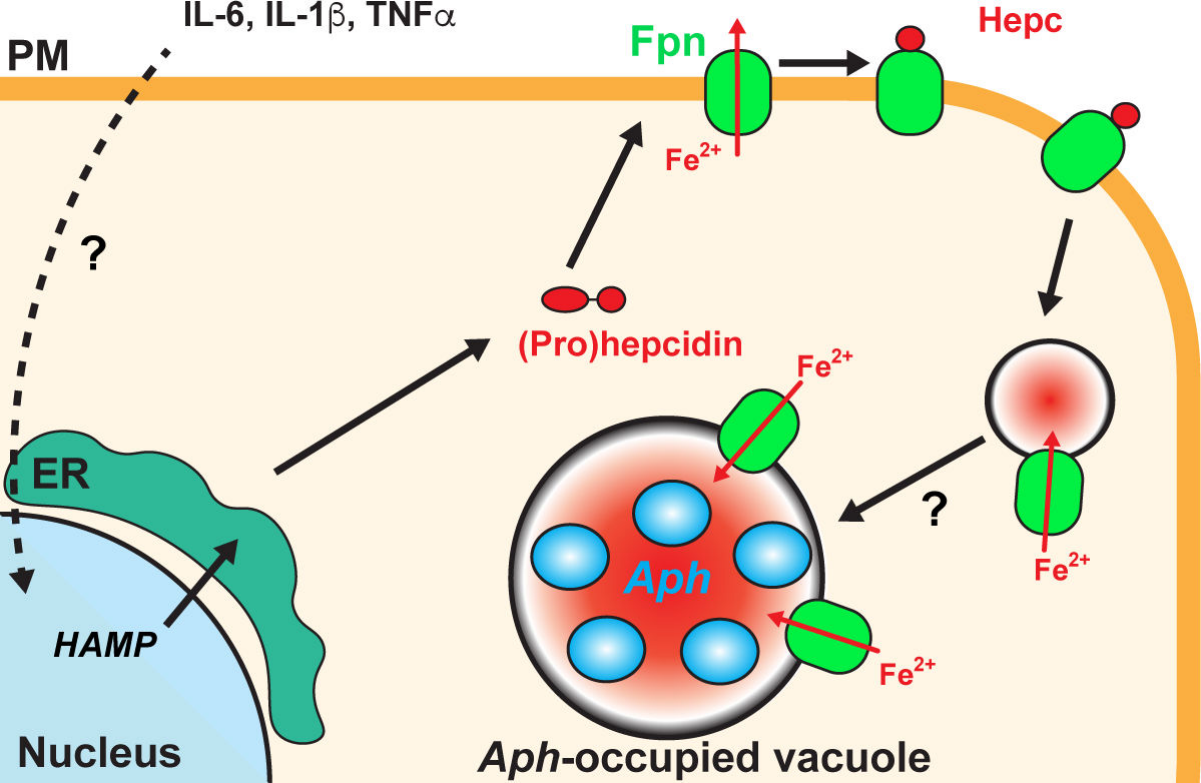
Hypothetical model: Infection-induced Hepc internalizes Fpn to *Aph* vacuole localization which facilitates iron uptake by *Aph.* PM Fpn exports iron from the cell. After *Aph* invasion, proinflammatory cytokines are upregulated concurrently with Hepc that induces endocytosis of Fpn, allowing Fpn endosomes to target *Aph-*vacuoles. Fpn localization on *Aph*-vesicles is vectorially positioned to transport Fe^2+^ from cytosolic sources into *Aph*-vacuole lumen, promoting bacterial growth. PM: plasma membrane, Fe^2+^: ferrous molecules, *Aph: Anaplasma phagocytophilum,* Fpn: ferroportin.

As Fpn domains required for localization to *Aph* vacuoles were those required for Fpn internalization in response to Hepc, which is encoded by *HAMP*, we examined if *Aph* infection induces *HAMP* expression. The RT-qPCR detected amplification of a low abundance of endogenous *HAMP* mRNA, which was elevated by *Aph* infection of RF/6A cells (Fig. 8A). As the primary cell type infected by *Aph* is granulocytes, we next examined *HAMP* mRNA in HL-60 cells. Here, *HAMP* mRNA was dramatically upregulated by *Aph* infection (Fig. 8B). Since *HAMP* translation produces prohepcidin which is processed to small active Hepc (57), we evaluated Hepc protein levels in uninfected or *Aph-*infected HL-60 cells. Western blot analysis using anti-human Hepc showed that *Aph* infection increases prohepcidin protein in HL-60 cells (similar size as in references 58, 59) (Fig. 8C and D). The *HAMP* gene is strongly upregulated in human hepatoma cells by the proinflammatory cytokine IL-6 (60); thus, we examined if *Aph* infection induces proinflammatory cytokines. Results showed IL-6, IL-1ß, and TNF⍺ mRNA were significantly upregulated after infection of HL-60 cells, with dramatic increase in IL-6 (Fig. 8E). These results altogether strongly indicate that *Aph-*induced inflammation subverts the Hepc/Fpn axis to encircle *Aph-*vacuoles with Fpn, which might export iron into vacuoles for *Aph* growth (Fig. 9).

## DISCUSSION

Fpn regulation is an important innate nutritional iron immune mechanism against intracellular infection. Increased expression of Fpn limits *Chlamydia psittaci, C. trachomatis*, or *Legionella pneumophila* infection (10). Fpn in the PM is co-internalized with *Staphylococcus aureus*, *Salmonella typhimurium,* or IgG-coated beads when phagocytosed by macrophages, but then rapidly (<15 min) removed from phagosomes and recycled back to the PM (61). In *Salmonella* infection of HeLa cells, some iron transport-competent Fpn remains on the *Salmonella*-containing vacuoles, functioning as a killing mechanism (28). Thus, these known Fpn-targeting host innate immune mechanisms against intracellular pathogens are (i) maintaining or increasing PM Fpn levels to reduce total intracellular Fe^2+^, (ii) removing Fpn from pathogen-containing phagosomes to prevent nutritional iron uptake, and/or (iii) selectively targeting Fpn to pathogen-containing phagosomes to inhibit infection by iron overload toxicity. In contrast, our current study revealed *Aph* subverts this nutritional immunity by recruiting Fpn to its replicative vacuole to promote its proliferation.

Subcellular localization of Fpn is variable. Most commonly, in HEK293 cells, *Xenopus* oocytes, MDCK cells, and hepatocytes, tagged-Fpn and endogenous Fpn are generally dispersed on the plasma membrane for export (17, 24, 51, 62). However, in duodenum enterocytes, Fpn localization is polarized to the basolateral side to release iron into circulation (23, 24, 62). Additionally, there are numerous reports of intracellular Fpn, such as in Kupffer cells, HEK293T cells, HK2 cells, and primary macrophages and macrophage cell lines, which implicate Fpn association with endosomal, lysosomal, and ER membranes (17, 24, 51, 63, 64). Fpn antibodies, membrane-permeabilization required for antibody staining, and Fpn-GFP transfection methods may also contribute to some of this variance. In this study, Fpn-GFP appeared on the PM of RF/6A epithelial cells and dispersed bright intracellular puncta. The 31A5 antibody (50), which recognizes a 5-residue epitope of the extracellular loop, stained both the RF/6A cells and HL-60 cells predominantly intracellularly. Additionally, while *HAMP* mRNA was detected in both cell types, it was upregulated by *Aph* infection ~10-fold greater in HL-60 than RF/6A cells. It is possible that HL-60 cells have a predisposition for intracellular vesicular Fpn which permits these cells to be readily infected by *Aph.* Indeed, HL-60 cell is the only mammalian cell line used successfully to culture isolate *Aph* from HGA patients’ blood (2, 49). In at least mouse macrophages, Fpn has been reported to be associated to lipid rafts, which is crucial for Fpn internalization (65). Notably, the *Aph*-vacuole is also enriched with proteins in lipid rafts in HL-60 and THP-1 cells (66, 67). Investigation of PM and intracellular Fpn trafficking across various cell types during infection will help further understanding *Aph* subversion of Fpn-mediated iron homeostasis and Fpn cell biology itself.

FerroFarRed is membrane-permeable and localizes in the endoplasmic reticulum (ER), making it to be advantageous for specific intracellular studies (44, 45). Both Fpn and LIP had granular appearance, suggesting they are in membrane-bound vesicles. When Fpn is endocytosed, vectorial orientation of iron transport is from cytoplasm to endosome lumen (Fig. 9). Thus, if Fpn endosomes fuse with *Aph*-vacuoles, iron is imported into the lumen of *Aph*-vacuoles (Fig. 9). Indeed, WT Fpn-GFP increased LIP in *Aph* vacuoles. We recently reported *Aph* recruits ER-Golgi exit sites to pathogen-occupied vacuoles using *Aph*-secreted protein, EgeA (38). Thus, a vesicular nature of FerroFarRed localized around *Aph* vacuoles may be retrograde-transported endosomes containing Fpn and ER-Golgi LIP.

Hepc decreases PM Fpn expression, consequently enhancing some intracellular pathogen infections. In macrophages infected with *Chlamydia psittaci, C. trachomatis*, or *Legionella pneumophila,* the addition of Hepc enhances their intracellular growth (10). On the other hand, protozoan *Leishmania donovanii* decreases Fpn protein levels in spite of upregulated mRNA and no induction of Hepc. Instead, *L. donovanii* represses *de novo* Fpn translation *via* FBXL5/Iron Regulatory Protein-2 (IRP) interactions with the Iron Responsive Element of Fpn mRNA (27). Some bacterial pathogens induce Hepc in macrophages and neutrophils *in vitro* and *in vivo* in a toll-like receptor 4 (TLR4)-dependent manner (22). However, undifferentiated HL-60 cells express low levels of TLR4 (68), and a defining trait of *Aph* is the absence of lipopolysaccharide (69), the ligand for TLR4. The LPS-TLR4 axis is, therefore, unlikely to directly drive Hepc production in *Aph*-infected HL-60 cells. Recently, it was reported that TLR5 or TLR2:TLR6 heterodimer activation in murine primary hepatocytes induces *HAMP* mRNA (70). Again, as *Aph* also lacks TLR5 ligand, flagella, or TLR6 ligand, diacyl peptides (36), and expression of TLR2 in undifferentiated HL-60 cells is low (68), these pathways may not be involved in *HAMP* mRNA upregulation in *Aph* infection.

Systemic *HAMP* gene transcriptional regulation involves signaling of iron levels and inflammation, both transcriptionally and post-transcriptionally. *HAMP* transcriptional activation requires the BMP/SMAD and HNF4α pathways and IL-6 activation of STAT3 (71–73) and stabilization of *HAMP* mRNA occurs through the HuR protein (74), but it is uncertain if these pathways are also involved locally in any infected cells. Likewise, pathogen-associated molecular patterns induce inflammation via TLR2/6 for downstream NF-κB crosstalk with Nrf2 to influence histone activity at the Fpn promoter, decreasing Fpn transcription which may increase the LIP available to pathogens (29). Clearly, though regulation of Hepc and Fpn can be advantageous for pathogens, there is no single universal model by which pathogens may accomplish this feat. Thus, more understanding of diverse mechanisms will be the key for developing *Aph-*specific or broadly effective therapeutics.

*In vitro* stimulation of fresh human hepatocytes with a panel of cytokines showed strong induction of Hepc mRNA by IL-6, but not IL-1α or TNF-α (75), indicating that IL-6 is a primary proinflammatory mediator of Hepc induction at a local level (60). Within 2 h after addition of *Aph*, IL-6, IL-1ß, and TNF-α mRNAs are induced in human peripheral blood leukocytes (PBLs) *in vitro* (76). In the present study, *Aph-*infection of HL-60 cells closely matched cytokine expression of infected human PBLs. Thus, Hepc upregulation in HL-60 cells by *Aph* infection may be a consequence of infection-induced inflammation and co-opted by *Aph* for Fpn internalization. Relatedly, ferritin acts as an acute-phase reactant, with levels rising sharply due to inflammation, infection, or cell damage, not only by iron overload (73, 77). The present study revealed *Aph* is able to upregulate cellular ferritin light chain protein in HL-60 cells in the landscape of IL-6 upregulation; thus, infection-induced inflammation may have multifaceted roles in iron availability to *Aph*. In contrast, a previous study reported diminished ferritin complex level in HL-60 cells after *Aph* infection, despite an increase in ferritin mRNA (primarily heavy chain), but also increases in ferritin complex and light chain mRNA in infected neutrophils (78). Reasons for these discrepancies are unclear. Interestingly, iron sensing by IRP-1 activity in human monocytic leukemia cell line THP-1 is unchanged by *Aph* infection (32), suggesting impaired intracellular iron sensing and regulation of iron metabolism by IRP-1 may also be an important contributing factor in *Aph* infection.

Both Fpn-GFP and endogenous Fpn infection did not drive all of the cellular Fpn to the *Aph-*vacuole. Thus, there is fine tuning and regulation for subversion of the inflammation-Hepc-Fpn axis that balances Fpn degradation, leaving a part of Fpn routed to—and sufficient for—the *Aph-*vacuole. Ultimately, this serves two purposes: to increase the stockpile of iron within the cell and to provide a means of transferring it across the *Aph-*vacuolar membrane. As Fpn does not co-localize or co-endocytose with extracellular *Aph* and *Aph* protein production is required to maintain colocalization*,* a distinct signal for Fpn internalization and targeting to *Aph* vacuoles is generated by viable intravacuolar bacteria.

## MATERIALS AND METHODS

### *Aph* and cell culture

Cultivation of *Aph* (HZ) in HL-60 cells (ATCC, Manassas, VA), preparation of host cell-free *Aph* via Dounce Homogenization, and infection were performed as described (49, 79). RF/6A monkey endothelial cells (ATCC) were cultured as described (43, 80). For experiments involving tetracycline, 5 µg/mL tetracycline (Sigma, St Lois, IL) was added to cultures at indicated timepoints.

### Plasmids

Plasmids encoding Fpn-GFP (WT) and Fpn-GFP mutants K8R, ∆229–269, ∆225–247, and ∆247–269 were previously described (20). For plasmids constructed in this study, primer sequences, and cloning strategies are described in Tables S1 and S2. Fpn-GFP mutants Y64H, D39A, and D181V were generated from Fpn-GFP (WT) using Q5 Site Directed Mutagenesis Kit (New England Biolabs, Ipswich, MA). Plasmids were purified with E.Z.N.A. Endo-free Plasmid DNA Mini Kit I (Omega Bio-tek, Norcross, GA), Endo-Free Maxi Kit (Qiagen, Hilden, Germany), or Nucleobond Midi EF Kit (Machery-Nagel, Düren, Germany).

### Antibodies

The mouse monoclonal antibody (mAb) 5C11 specific to *Aph* P44 and anti-EgeA were described previously (38, 47). Horse antiserum immunoreactive with *Aph* (anti-*Aph)* from was preadsorbed with uninfected cell culture before use. The mouse anti-human Fpn (31A5) antibody was provided by Amgen, Inc. (Thousand Oaks, CA). The following antibodies were obtained from commercial sources—Santa Cruz Biotechnology (Dallas, TX): mouse anti-human β-actin (C4); Novus Biologicals (Centennial, CO): rabbit anti-human Fpn (21502); Alpha Diagnostics International (San Antonio, TX): rabbit anti-human Hepc (HEPC13-S). Secondary antibodies including Alexa Fluor 488 (AF488)-conjugated goat anti-mouse IgG and AF555-conjugated goat anti-rabbit IgG were obtained from Thermo Fisher, Cy3-conjugated goat anti-horse Ig was obtained from Jackson Immunoresearch, and peroxidase-labeled goat anti-mouse or anti-rabbit secondary antibodies were obtained from Seracare (Milford, MA).

### RT-qPCR

At indicated times post infection, cells were washed with phosphate-buffered saline (PBS; 8 mM Na_2_HPO_4_, 1.47 mM KH_2_PO_4_, 2.67 mM KCl, 137.9 mM NaCl, pH 7.4), and RNAs were extracted with the RNeasy Mini kit (Qiagen, Germantown, MD). cDNA was synthesized using the Maxima H minus First Strand cDNA Synthesis kit with Random Hexamer primers (Thermo Fisher Scientific, Waltham, MA), and bacterial and host gene expressions were determined by RT-qPCR with specific primers for *Aph* 16S rRNA (81), human genes *HPRT* (this study), *HAMP* (82), *TNF⍺* (83), *IL-1ß* (84), and IL-6 (85) (Table S2), using Maxima SYBR Green/ROX Master Mix (Thermo Fisher Scientific) in an AriaMx Real-time PCR system (Agilent, Santa Clara, CA) according to the manufacturers’ instructions.

### Western blot analysis

Uninfected or *Aph*-infected cells at 2 dpi were washed with PBS, and soluble protein was extracted using commercial mammalian protein extraction reagent (M-PER, ThermoFisher Scientific). Following high speed centrifugation, soluble protein was reduced with 5 × loading buffer (30% Glycerol, 2.3 M 2-mercaptoethanol, 4% [wt/vol] SDS, 0.03% bromophenol blue, 250 mM Tris-HCl pH 6.8) and denatured by boiling for 5 min. Proteins were separated by 10% (Actin and P44), 12% (FTL), or 15% (Hepc) SDS-PAGE gel electrophoresis followed by semi-dry transfer to 0.45 µm nitrocellulose (actin, P44, and Hepc) or methanol-activated 0.2 µm PVDF (FTL). Protein loads were normalized by BCA Assay (Pierce, Rockford, IL) and verified by Ponceau S staining of the membrane and anti-actin immunoblots. Membranes were blocked and blotted with 5% skim milk and 0.05% Tween-20 in Tris-buffered saline (TBS, 15 mM NaCl and 5 mM Tris, pH 7.4) and developed with ECL Detection Reagent (ThermoFisher Scientific). Chemiluminescence was photographed with a GE Amersham Imager 680 (GE Healthcare, Chicago, IL) and band densities recorded in NIH ImageJ.

### Transfection, IF labeling, and cellular localization analysis

RF/6A cells adhered to coverslips were transfected using FuGENE HD reagent (Promega, Madison, WI) with plasmids at 3:1 ratio (3 μL FuGENE HD:1 μg plasmids) in a 12-well plate according to the manufacturer’s instructions. Cells were then infected with host cell free *Aph* at MOI 500 at timepoints indicated. In some experiments, cells were infected by co-culture with highly infected HL-60 cells for 1 day prior to transfection. For IF labeling, cells were fixed and permeabilized with ice cold 80% methanol/20% acetone and labeled with primary then fluorescently conjugated secondary antibodies diluted in 0.05% bovine serum albumin (BSA, Sigma-Aldrich). In some experiments, cells were fixed with 4% paraformaldehyde (Sigma) and labeled with primary and then fluorescence-conjugated secondary antibodies diluted in PBS supplemented with 0.1% gelatin (Bio-Rad, Hercules, CA), 0.05% BSA, and 0.1% saponin (Sigma) (PGBS). Uninfected HL-60 cells or *Aph-*infected HL-60 cells were cytocentrifuged onto a microscope slide, fixed with 4% paraformaldehyde, and permeabilized with PGBS. Double immuno-labeling was carried out by successive staining in PGBS with anti-Fpn 31A5 primary and secondary anti-mouse IgG antibodies, then primary anti-*Aph* EgeA and secondary anti-rabbit IgG antibodies. In all experiments, the host cell nuclei and *A. phagocytophilum* DNA were labeled with Hoechst 33342 (Thermo Fisher Scientific).

### LIP analysis

For live cell LIP analysis, RF/6A cells were adhered onto glass-bottom tissue culture dishes (CellVis, Mountain View, CA) and infected with host cell-free *Aph* for 2 days. LIP was detected after incubation with 5 µM FerroFarRed dye (Sigma-Aldrich) for 1 h at 37°C with 5% CO_2_ using an Oko-Labs (Pozzuoli, NA, Italy) incubation chamber microscope mount. Nuclei and *Aph* were labeled with Hoechst, and then live cells were observed under Leica Thunder Imager at 37°C (35). For LIP analysis of *Aph* occupying Fpn(WT)-GFP, Fpn(D39A)-GFP, and Fpn(D181V)-GFP-transfected RF/6A cells were infected with host cell-free *Aph* (MOI: 500) at 2 dpt (day post transfection). At 1.5 dpi (3.5 dpt), cells were incubated with 5 μM FerroFarRed at 37°C for 1 h.

### Image analysis

Fluorescence images with overlaid differential interference contrast (DIC) images were acquired under the same settings among experimental groups and analyzed with a Leica Thunder imaging system with computational clearing to remove any out-of-focus background (Leica Microsystems, Deerfield, IL). NIH ImageJ software was used to measure line scan relative signal intensities or area mean fluorescent intensities. A macro (described in Fig. S1) was written to randomly locate and calculate the area of a defined ROI dimensions of each *Aph*-vacuole to measure FerroFarRed mean fluorescence intensity (MFI) of “non-vacuoles” from infected or uninfected cells within the same field of view.

### Statistical analysis

Statistical analysis was performed with Student’s unpaired *t* test or a one-way analysis of variance (ANOVA). *P* < 0.05 was considered statistically significant. All statistical analyses were performed using Prism 10 (GraphPad, La Jolla, CA).

## Data Availability

Data will be made publicly available upon publication and upon request for peer review.

## SUPPLEMENTAL MATERIAL

Supplemental Material mbio.01134-26-s0001.docx Fig. S1-S5; Tables S1 and S2.

ASM does not own the copyrights to Supplemental Material that may be linked to, or accessed through, an article. The authors have granted ASM a non-exclusive, world-wide license to publish the Supplemental Material files. Please contact the corresponding author directly for reuse.
