# Supplementary material for: Anaplasma phagocytophilum subversion of host hepcidin-ferroportin iron nutritional immunity
Source: mBio. 2026 Jun 15;17(7):e01134-26. doi: 10.1128/mbio.01134-26 (PMC13344017; doi:10.1128/mbio.01134-26)
Supplement: Supplemental Material — Fig. S1-S5; Tables S1 and S2. [file mbio.01134-26-s0001.docx]

# Supplementary Information for:

# *Anaplasma phagocytophilum* Subversion of Host Hepcidin-Ferroportin Iron Nutritional Immunity

# ^1^Stephen L. Denton, ^1^Mingqun Lin, ^2^Elizabeta Nemeth, and ^1^Yasuko Rikihisa#

# ^1^Department of Veterinary Biosciences, College of Veterinary Medicine, Infectious Diseases Institute, The Ohio State University, Columbus, OH

^2^David Geffen School of Medicine, University of California at Los Angeles, Los Angeles, CA

# Running Title: Ferroportin manipulation by *Anaplasma*

# KEYWORDS: *Anaplasma phagocytophilum*, ferroportin, iron transport, hepcidin

# # Address correspondence to Yasuko Rikihisa, [rikihisa.1@osu.edu](mailto:rikihisa.1@osu.edu)

#
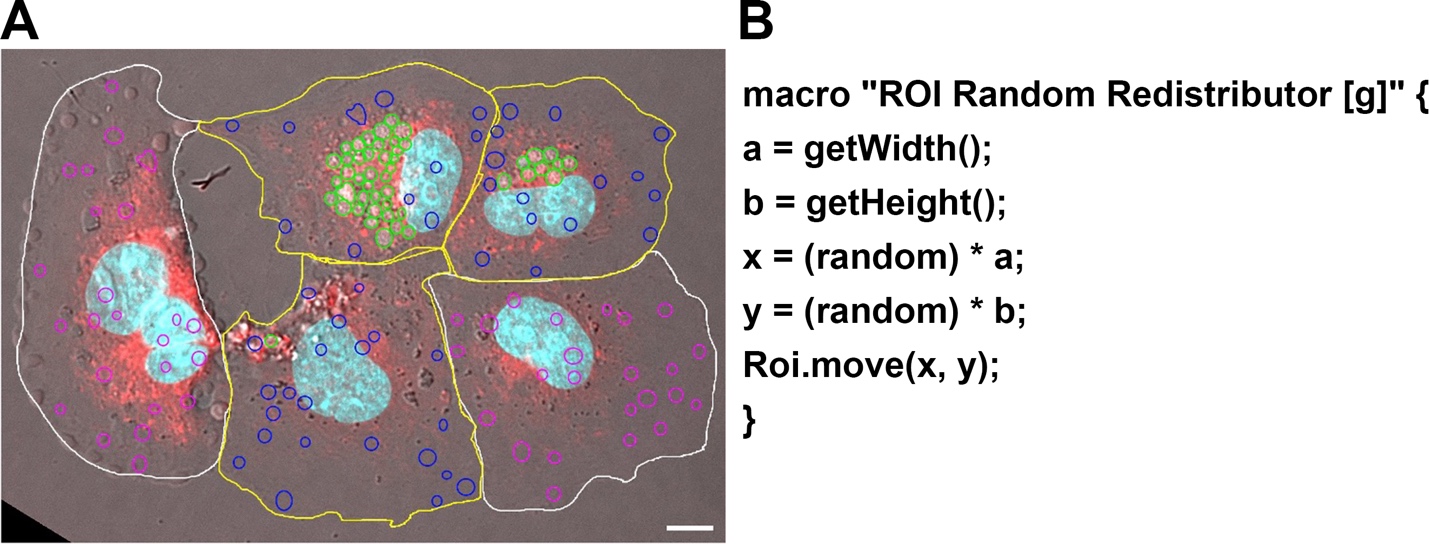
Figure S1. Methodological Description of LIP measurements.

For experiments of fluorescent measurement of the labile iron pool (LIP) in live cells, images were collected and FerroFarRed mean fluorescent intensity (MFI) of regions of interest (ROI) were measured with ImageJ (NIH). A) Example ROI demarcation in a merged Hoechst, FerroFarRed, and DIC image. For cell measurements (Fig. 1B), cell boundaries were determined in the DIC channel and classified as uninfected (white boundary) or *Aph*-infected, by the presence of at least one non-nuclear vacuole containing bacterial DNA (yellow boundary). For vacuole measurements (Fig. 1B), within infected cells the boundaries of *Aph-*vacuoles were determined by round bacterial DNA clusters in the Hoechst channel (green). For each *Aph-*vacuole, the ROI Random Redistributor macro (shown in panel B), was applied to move the exact dimensions of the ROI to random locations in the image. If the random redistribution was contained within an infected cell boundary, it was classified as an infected cell non-vacuole (blue), and if it fell into an uninfected cell boundary, it was classified as an uninfected cell non-vacuole (magenta). All MFI measurements were taken in the FerroFarRed channel.

**
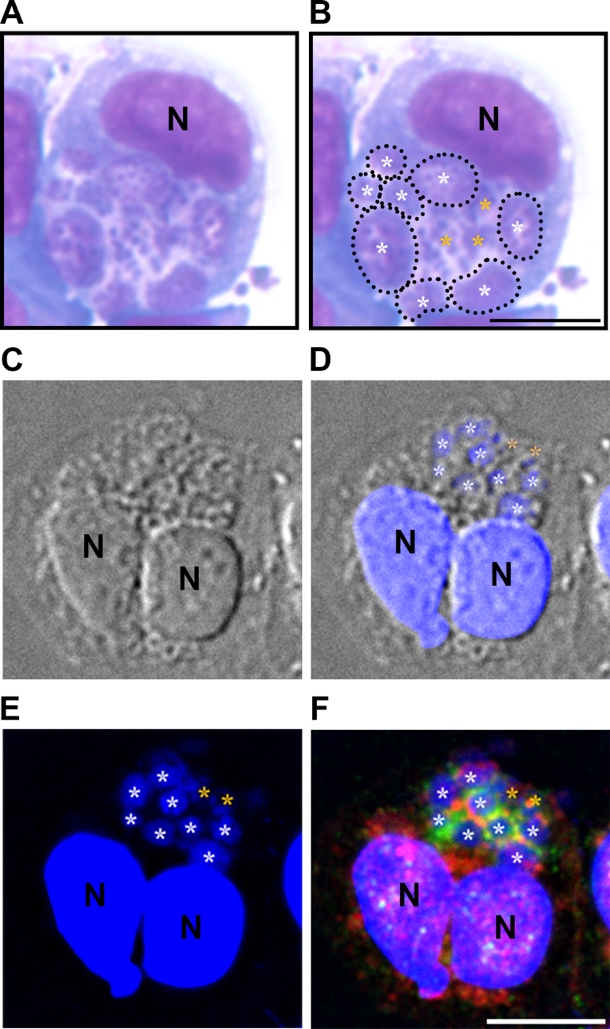
**

**Figure S2. Identification of *Aph-*vacuoles.**

Clusters of non-nuclear DNA in *Aph-*infected cells can identify *Aph*-vacuoles. All images are representative of HL-60 cells infected with *Aph* for two days. **A-B)** Unmarked (A) and annotated (B) Hema3 staining of HL-60 cell infected with *Aph* at 2 dpi show detailed cell morphology. White asterisks indicate obviously clustered *Aph* bacteria within an *Aph*-vacuole. Orange asterisks indicate ambiguous clusters of *Aph* bacteria or singular bacteria isolated to individual vacuoles. **C-F)** Images of *Aph-*infected HL-60 cells as in Fig. 2E including (C) Differential interference contrast (DIC), (D) Hoechst (blue) merged with DIC, (E) Hoechst only, and (F) Hoechst merged with EgeA (red), Fpn (31A5, green). Asterisks as in A and B. Nucleus, N, scale bar, 10 µm (white).


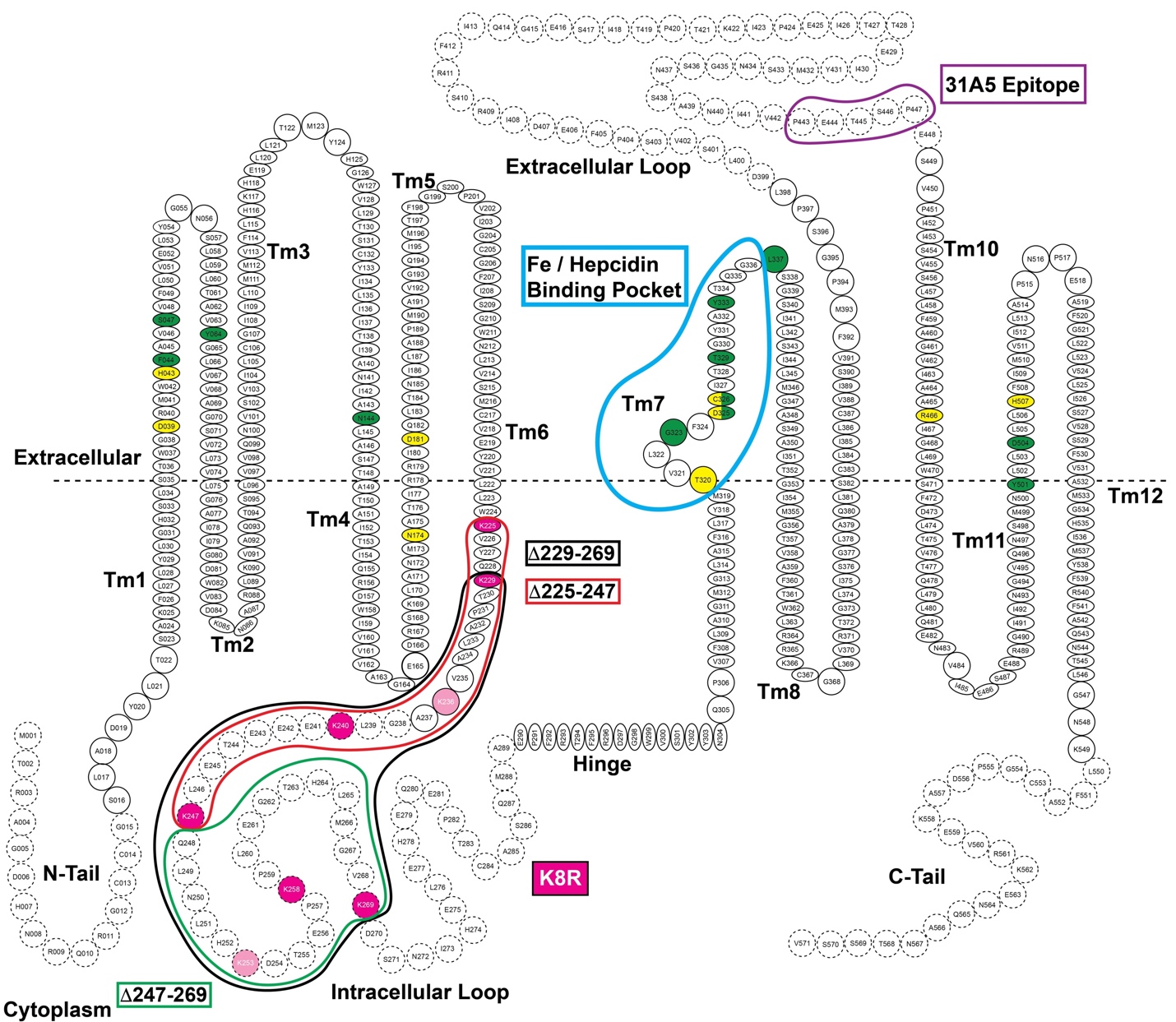


**Figure S3. 2-Dimensional representation of Fpn structure interpreted from Protein Database.**

Illustration adapted from concept in ([1](#_ENREF_1)) and based on Protein Database (PBD) crystal structures 5AYN, 5AYO, 8C03, 8BZY, 6W4S, 6WBV, and 6W4V ([2-4](#_ENREF_2)). Amino acids with dotted lines have not been resolved in reported crystal structures, whereas solid lines have been resolved and flattened circles represent alpha helical structure. Yellow designates residues that are involved in iron binding or export function ([5-7](#_ENREF_5)), and green designates residues involved in Hepc binding ([8](#_ENREF_8)) ([1](#_ENREF_1), [9-11](#_ENREF_9)). Pink residues indicate lysines that have been mutated in the K8R multiple mutation of Fpn-GFP and intensely colored if ubiquitinated by hepcidin ([9](#_ENREF_9)). Circled regions have been deleted in Fpn-GFP mutants and are color matched to the residue number([1](#_ENREF_1)). The epitope of antibody 31A5 is shown.

**
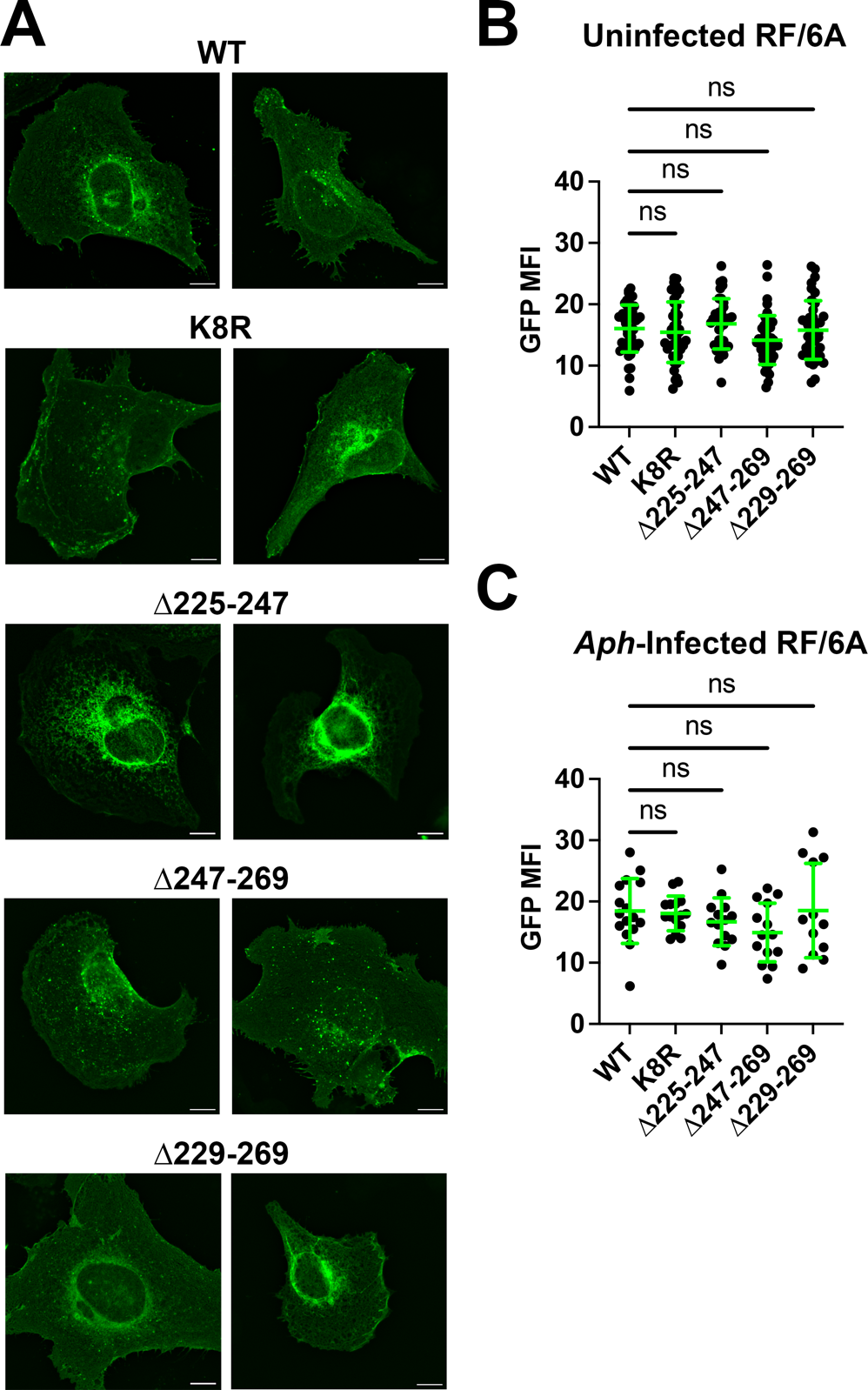
**

**Figure S4. Fpn-GFP deletion mutant expression and morphology in uninfected RF/6A cells.**

**(A)** Fpn-GFP plasmids (WT, K8R, ∆229-269, ∆225-247, and ∆247-269) were transfected into RF/6A cells and harvested at 2 dpt. White bar, 10 µm. (**B)** Mean GFP Fluorescence Intensity of cells in (A) expressing each plasmid. N = 38-49 cells for each plasmid. **(C)** Mean GFP Fluorescence Intensity of transfected and infected cells as in Main Figure 5 expressing each plasmid. N = 12-16 cells for each plasmid. Statistics: Ordinary One-Way ANOVA where ns indicates p > 0.05.


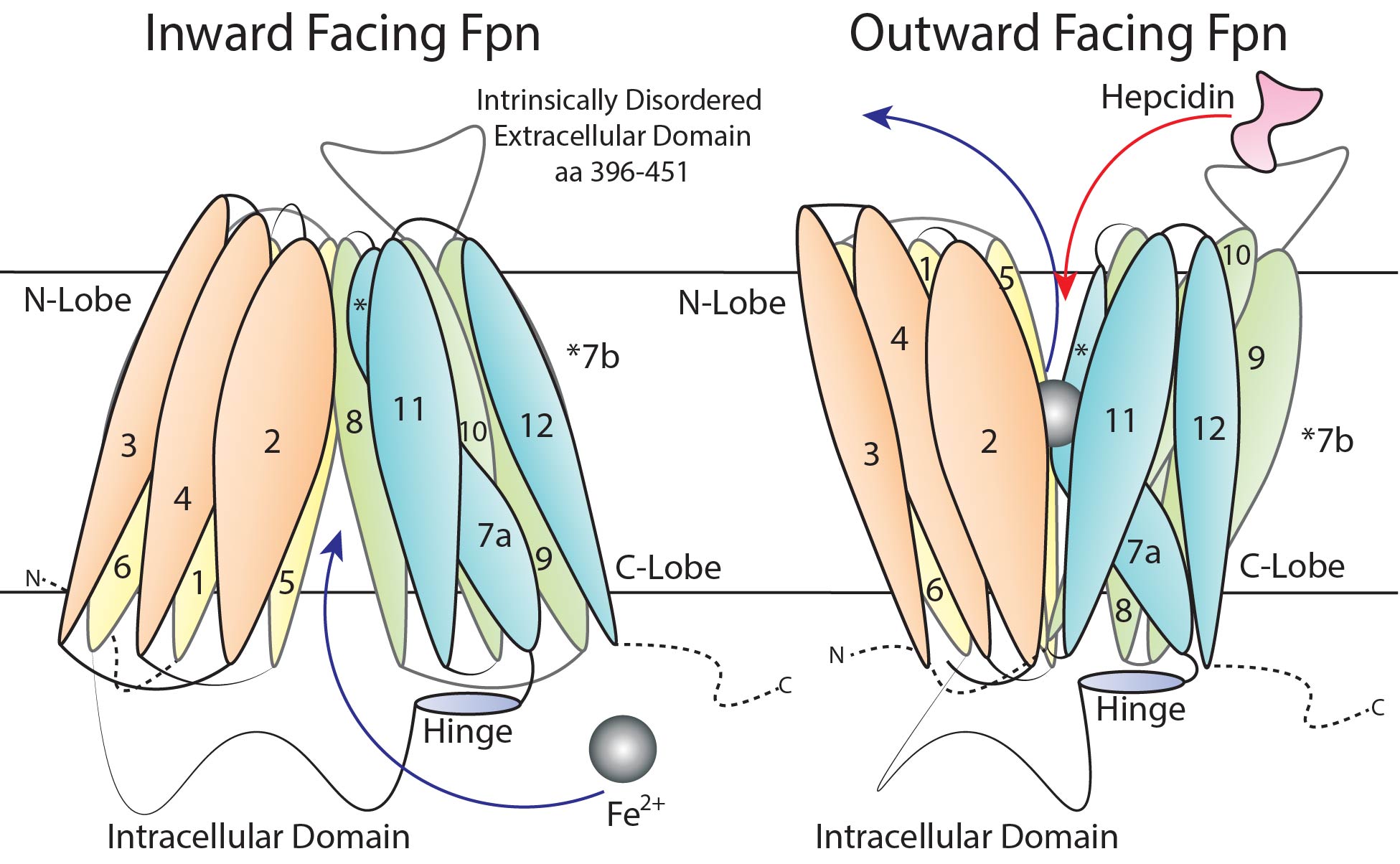


**Figure S5. Molecular mechanism of ferroportin-mediated iron export and hepcidin occlusion.**

Illustration interpreted from structural and functional analysis reports ([1-11](#_ENREF_1)).

Fpn is made up by twelve alpha-helical transmembrane domains (TM1-12) divided into two distinct lobes according to their amino and carboxyl termini, named the N-Lobe (TM1-6, orange and yellow, colored to differentiate TM domains) and the C-Lobe (TM7-12, blue and green). Further features include a perpendicularly oriented alpha helical “hinge” anterior to TM7, a disordered intracellular domain between the hinge and TM6, and a disordered extracellular domain between TM9 and TM10. Ferroportin is crystalized in two distinct conformations according to the exposed orientation of the iron/hepcidin-binding pocket: the inward-facing state and the outward facing state ([2-4](#_ENREF_2)). In both conformational states, the transmembrane domains are divided into two lobes, according to their amino and carboxyl termini orientation: the N-Lobe made up of interweaving TM1-6 (colored orange and yellow) and the C-Lobe made up of interweaving TM7-12 (colored blue and green). Conformational states differ dramatically in positioning of the two alpha helices comprising TM7, denoted as TM7a and TM7b. Accordingly, TM7 is heavily composed of residues critical for iron-binding, export, and hepcidin binding ([10](#_ENREF_10), [11](#_ENREF_11)). Intracellular ferrous iron has access to the inward-facing state of ferroportin, which triggers a ligand-induced conformational change to the outward-facing state, opening the binding pocket to the extracellular environment for iron export and hepcidin binding involving interactions of other transmembrane domains TM1 (including aa D39 and Y64), TM5 (including aa D181), and TM10 ([3](#_ENREF_3), [10](#_ENREF_10)). Hepcidin binding ferroportin in the outward facing state both occludes the pocket, preventing iron export, as well as initiating the ubiquitination-internalization-degradation cascade ([1](#_ENREF_1), [9](#_ENREF_9), [12](#_ENREF_12)).

| **Plasmid Name** | **Description** | **Reference** |
| --- | --- | --- |
| Fpn-GFP (WT) | Functional Human Fpn with C-Terminal EGFP Tag. | ([10](#_ENREF_10)) |
| Fpn-GFP (∆229-269) | C-terminal EGFP-tagged Fpn lacking residues 229-269 that make up the intracellular loop. Kanamycin selection. | ([1](#_ENREF_1)) |
| Fpn-GFP (∆225-247) | C-Terminal EGFP-tagged Fpn lacking residues 225-249 that make up the intracellular loop | ([1](#_ENREF_1)) |
| Fpn-GFP (∆247-269) | C-Terminal EGFP-tagged Fpn lacking residues 249-269 that make up the intracellular loop | ([1](#_ENREF_1)) |
| Fpn-GFP (K8R) | C-Terminal EGFP-tagged Fpn where the 8 lysine residues of the intracellular loop have been mutated to arginine. | ([9](#_ENREF_9)) |
| Fpn-GFP (Y64H) | C-Terminal EGFP-tagged Fpn where the tyrosine residue position 64 was mutated to histidine using whole-plasmid PCR and ligation (Q5 SDM, NEB) | ([8](#_ENREF_8), [10](#_ENREF_10)) |
| Fpn-GFP (D39A) | C-Terminal EGFP-tagged Fpn where the aspartic acid residue position 39 was mutated to alanine using whole-plasmid PCR and ligation (Q5 SDM, NEB) | This study, ([6](#_ENREF_6)) |
| Fpn-GFP (D181V) | C-Terminal EGFP-tagged Fpn where the aspartic acid residue position 181 was mutated to valine using whole-plasmid PCR and ligation (Q5 SDM, NEB) | This study, ([5](#_ENREF_5), [6](#_ENREF_6)) |

**Table S1. List of plasmids used in this study.**

Plasmids used in the study are listed by name as it appears in the article. Plasmid descriptions include properties of the plasmid and/or their construction by Q5® site-directed mutagenesis (Q5 SDM) using New England Biolabs (NEB) according to manufacturer’s protocol. References for originating article or articles describing the functionality of the residues of the domain are numbered for each plasmid according to this Supplementary Information References.

| **Target Gene** | **Purpose** | **Primer Sequence (5' to 3')** | **Product Size (bp)** | **Reference** |
| --- | --- | --- | --- | --- |
| Human *Fpn* mRNA | RT-qPCR | F: CGTCATTGCTGCTAGAATCG | 203 | ([13](#_ENREF_13)) |
|  |  | R: AGACTGAAATCAATACGAGC |  |  |
| Human *HPRT* mRNA | RT-qPCR | F: CCCTGGCGTCGTGATTAGTG | 190 | This study |
|  |  | R: GAGCACACAGAGGGCTACAA |  |  |
| *Aph* 16S rRNA | RT-qPCR | F: GGTGAGTAATGCATAGGAATC | 108 | ([14](#_ENREF_14)) |
|  |  | R: GCTCATCTAATAGCGATAAATC |  |  |
| Human *HAMP* | RT-qPCR | F: TCCCACAACAGACGGGACAA | 138 | ([15](#_ENREF_15)) |
|  |  | R: AGCAGCCGCAGCAGAAAAT |  |  |
| Human IL-1β | RT-qPCR | F: ATGCACCTGTACGATCACTGA | 142 | ([16](#_ENREF_16)) |
|  |  | R: ACAAAGGACATGGAGAACACC |  |  |
| Human IL-6 | RT-qPCR | F: GGAGACTTGCCTGGTGAAAA | 181 | ([17](#_ENREF_17)) |
|  |  | R: GTCAGGGGTGGTTATTGCAT |  |  |
| Human TNFɑ | RT-qPCR | F: TCAGCCTCTTCTCCTTCCTG | 124 | ([18](#_ENREF_18)) |
|  |  | R: GCCAGAGGGCTGATTAGAGA |  |  |
| SDM of Fpn-GFP (WT) for **Y64H** Mutation | Cloning (SDM) | F: GACAGCAGTCCATGGGCTGGTGG |  | This study, ([8](#_ENREF_8), [10](#_ENREF_10)) |
|  |  | R: AAAAGGAGGCTGTTTCCATAG |  |  |
| SDM of Fpn-GFP (WT) for **D39A** Mutation | Cloning (SDM) | F: TACTTGGGGAGCGCGGATGTGGC |  | This study, ([6](#_ENREF_6)) |
|  |  | R: GAGAGAGAATGACCAAGGTAG |  |  |
| SDM of Fpn-GFP (WT) for **D181V** Mutation | Cloning (SDM) | F: ACGAAGGATTGTGCAGTTAACCAAC |  | This study, ([5](#_ENREF_5), [6](#_ENREF_6)) |
|  |  | R: ATTGTGGCATTCATATTTG |  |  |
| Underlined: Sequence for desired mutations | | |  |  |

**Table S2. List of primers used in this study.**

Primer pair sequences used in the study are listed by name according to the targeted amplification of the PCR product. The purpose for each primer pair is listed for plasmid construction (Cloning), including site-directed mutagenesis (SDM) and measurements of mRNA abundance by reverse transcriptase quantitative polymerase chain reaction (RT-qPCR). References indicate originating article of primer pair sequence design according to this Supplementary Information References.

**References**

1. Qiao B, Sugianto P, Fung E, Del-Castillo-Rueda A, Moran-Jimenez MJ, Ganz T, Nemeth E. 2012. Hepcidin-induced endocytosis of ferroportin is dependent on ferroportin ubiquitination. Cell Metab 15:918-24.

2. Taniguchi R, Kato HE, Font J, Deshpande CN, Wada M, Ito K, Ishitani R, Jormakka M, Nureki O. 2015. Outward- and inward-facing structures of a putative bacterial transition-metal transporter with homology to ferroportin. Nat Commun 6:8545.

3. Billesbolle CB, Azumaya CM, Kretsch RC, Powers AS, Gonen S, Schneider S, Arvedson T, Dror RO, Cheng Y, Manglik A. 2020. Structure of hepcidin-bound ferroportin reveals iron homeostatic mechanisms. Nature 586:807-811.

4. Lehmann EF, Liziczai M, Drozdzyk K, Altermatt P, Langini C, Manolova V, Sundstrom H, Durrenberger F, Dutzler R, Manatschal C. 2023. Structures of ferroportin in complex with its specific inhibitor vamifeport. Elife 12.

5. Majore S, Bonaccorsi di Patti MC, Valiante M, Polticelli F, Cortese A, Di Bartolomeo S, De Bernardo C, De Muro M, Faienza F, Radio FC, Grammatico P, Musci G. 2018. Characterization of three novel pathogenic SLC40A1 mutations and genotype/phenotype correlations in 7 Italian families with type 4 hereditary hemochromatosis. Biochim Biophys Acta Mol Basis Dis 1864:464-470.

6. Bonaccorsi di Patti MC, Polticelli F, Cece G, Cutone A, Felici F, Persichini T, Musci G. 2014. A structural model of human ferroportin and of its iron binding site. FEBS J 281:2851-60.

7. Pan Y, Ren Z, Gao S, Shen J, Wang L, Xu Z, Yu Y, Bachina P, Zhang H, Fan X, Laganowsky A, Yan N, Zhou M. 2020. Structural basis of ion transport and inhibition in ferroportin. Nat Commun 11:5686.

8. Raszeja-Wyszomirska J, Caleffi A, Milkiewicz P, Pietrangelo A. 2014. Ferroportin-related haemochromatosis associated with novel Y64H mutation of the SCL40A1 gene. Prz Gastroenterol 9:307-9.

9. Aschemeyer S, Qiao B, Stefanova D, Valore EV, Sek AC, Ruwe TA, Vieth KR, Jung G, Casu C, Rivella S, Jormakka M, Mackenzie B, Ganz T, Nemeth E. 2018. Structure-function analysis of ferroportin defines the binding site and an alternative mechanism of action of hepcidin. Blood 131:899-910.

10. Fernandes A, Preza GC, Phung Y, De Domenico I, Kaplan J, Ganz T, Nemeth E. 2009. The molecular basis of hepcidin-resistant hereditary hemochromatosis. Blood 114:437-43.

11. Schimanski LM, Drakesmith H, Merryweather-Clarke AT, Viprakasit V, Edwards JP, Sweetland E, Bastin JM, Cowley D, Chinthammitr Y, Robson KJ, Townsend AR. 2005. In vitro functional analysis of human ferroportin (FPN) and hemochromatosis-associated FPN mutations. Blood 105:4096-102.

12. Nemeth E, Tuttle MS, Powelson J, Vaughn MB, Donovan A, Ward DM, Ganz T, Kaplan J. 2004. Hepcidin regulates cellular iron efflux by binding to ferroportin and inducing its internalization. Science 306:2090-3.

13. Kondaiah P, Aslam MF, Mashurabad PC, Sharp PA, Pullakhandam R. 2019. Zinc induces iron uptake and DMT1 expression in Caco-2 cells via a PI3K/IRP2 dependent mechanism. Biochem J 476:1573-1583.

14. Xiong Q, Lin M, Huang W, Rikihisa Y. 2019. Infection by Anaplasma phagocytophilum Requires Recruitment of Low-Density Lipoprotein Cholesterol by Flotillins. MBio 10.

15. Detivaud L, Nemeth E, Boudjema K, Turlin B, Troadec MB, Leroyer P, Ropert M, Jacquelinet S, Courselaud B, Ganz T, Brissot P, Loreal O. 2005. Hepcidin levels in humans are correlated with hepatic iron stores, hemoglobin levels, and hepatic function. Blood 106:746-8.

16. Gicquel T, Robert S, Loyer P, Victoni T, Bodin A, Ribault C, Gleonnec F, Couillin I, Boichot E, Lagente V. 2015. IL-1beta production is dependent on the activation of purinergic receptors and NLRP3 pathway in human macrophages. FASEB J 29:4162-73.

17. Ouhara K, Munenaga S, Kajiya M, Takeda K, Matsuda S, Sato Y, Hamamoto Y, Iwata T, Yamasaki S, Akutagawa K, Mizuno N, Fujita T, Sugiyama E, Kurihara H. 2018. The induced RNA-binding protein, HuR, targets 3'-UTR region of IL-6 mRNA and enhances its stabilization in periodontitis. Clin Exp Immunol 192:325-336.

18. Pinto JP, Dias V, Zoller H, Porto G, Carmo H, Carvalho F, de Sousa M. 2010. Hepcidin messenger RNA expression in human lymphocytes. Immunology 130:217-30.
